# Plastic Rewiring of Sef1 Transcriptional Networks and the Potential of Nonfunctional Transcription Factor Binding in Facilitating Adaptive Evolution

**DOI:** 10.1093/molbev/msab192

**Published:** 2021-06-27

**Authors:** Po-Chen Hsu, Tzu-Chiao Lu, Po-Hsiang Hung, Yu-Ting Jhou, Ahmed A A Amine, Chia-Wei Liao, Jun-Yi Leu

**Affiliations:** 1 Institute of Molecular Biology, Academia Sinica, Taipei, Taiwan, ROC; 2 Research Center for Healthy Aging and Institute of New Drug Development, China Medical University, Taichung, Taiwan, ROC; 3 Institute of Biochemistry and Molecular Biology, National Yang Ming Chiao Tung University, Taipei, Taiwan, ROC; 4 Genome and Systems Biology Degree Program, National Taiwan University and Academia Sinica, Taipei, Taiwan, ROC; 5 Molecular and Cell Biology Program, Taiwan International Graduate Program, Academia Sinica and Graduate Institute of Life Science, National Defense Medical Center, Taipei, Taiwan, ROC

**Keywords:** transcriptional rewiring, nonfunctional transcription factor binding, TCA cycle regulation, transcriptional network evolution, phenotypic diversity, *Lachancea kluyveri*

## Abstract

Prior and extensive plastic rewiring of a transcriptional network, followed by a functional switch of the conserved transcriptional regulator, can shape the evolution of a new network with diverged functions. The presence of three distinct iron regulatory systems in fungi that use orthologous transcriptional regulators suggests that these systems evolved in that manner. Orthologs of the transcriptional activator Sef1 are believed to be central to how iron regulatory systems developed in fungi, involving gene gain, plastic network rewiring, and switches in regulatory function. We show that, in the protoploid yeast *Lachancea kluyveri*, plastic rewiring of the *L. kluyveri* Sef1 (Lk-Sef1) network, together with a functional switch, enabled Lk-Sef1 to regulate TCA cycle genes, unlike *Candida albicans* Sef1 that mainly regulates iron-uptake genes. Moreover, we observed pervasive nonfunctional binding of Sef1 to its target genes. Enhancing Lk-Sef1 activity resuscitated the corresponding transcriptional network, providing immediate adaptive benefits in changing environments. Our study not only sheds light on the evolution of Sef1-centered transcriptional networks but also shows the adaptive potential of nonfunctional transcription factor binding for evolving phenotypic novelty and diversity.

## Introduction

Altered transcriptional regulation is thought to play a crucial role in the adaptive evolution of various organisms (Carroll 2000; Wagner and Lynch 2008; Romero et al. 2012). In animals and plants, many morphological innovations in the body plan arose from changes to *cis*-regulatory elements (Wilson and Odom 2009; Peter and Davidson 2011; Della Pina et al. 2014). Similar changes also contribute to speciation and adaptive evolution in microorganisms, which do not have complex developmental programs (Perez and Groisman 2009; Li and Johnson 2010; Wang et al. 2011). Although *cis*-element changes are well documented as influencing expressional divergence between species (Wittkopp et al. 2004; Tirosh et al. 2009), modifications to transcriptional regulation often involve coordination between *cis*-regulatory elements and *trans*-acting regulatory factors (Wagner and Lynch 2008). Both these elements need to be taken into consideration in order to understand the tempo and mode of regulatory evolution. 

“Transcriptional rewiring” is a term used to describe the evolution of transcriptional regulation across species by changing the *cis*-regulatory elements in promoters or by replacing the *trans*-acting regulatory factors in a gene regulatory network (Scannell and Wolfe 2004; Dalal and Johnson 2017). By dissecting both *cis*-elements and *trans*-factors among different species, studies in yeasts have shown that large-scale rewiring has occurred in various cellular pathways including those underlying mating-type determination, ribosomal protein production, sugar metabolism, nucleotide metabolism, meiosis, and sporulation (Tsong et al. 2003; Dalal and Johnson 2017). Interestingly, generally consistent patterns of gene expression are often preserved after extensive rewiring in the regulatory networks, suggesting that the rewiring events might be caused by genetic drift and then accompanied by compensatory evolution to maintain the essential functions of the pathways (Nocedal et al. 2017). On the other hand, a functional switch of the *trans*-acting regulatory factor may occur after the plastic rewiring of transcriptional networks, leading to the formation of an alternative network with new functions (Nocedal et al. 2017). Currently, it remains unclear whether some novel phenotypic outcomes are also generated (as a byproduct) during the process of large-scale rewiring that might endow organisms with novel phenotypic adaptations to changing environments.

**Fig. 1.**
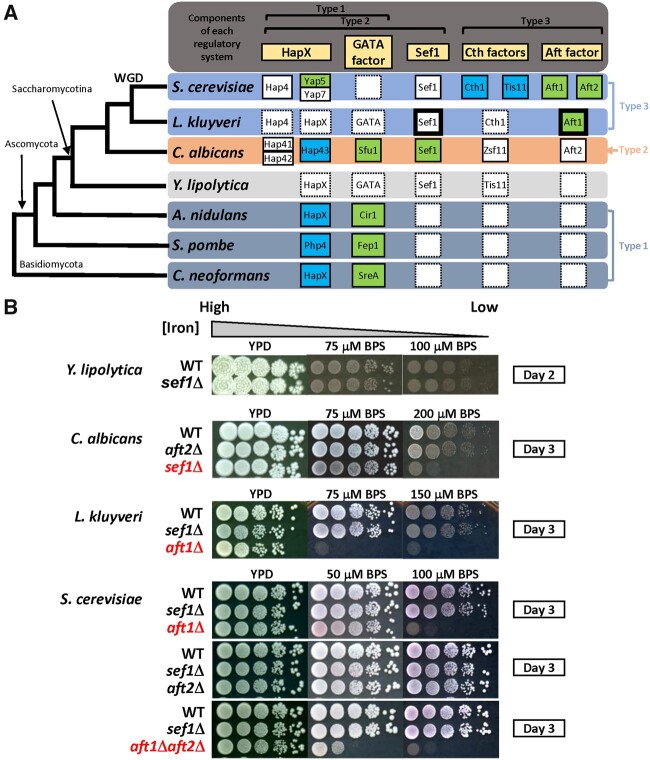
Potential components of iron-regulatory networks in yeast species and the growth of *sef1*Δ mutants under iron-deprived conditions. (*A*) Simplified phylogenetic tree for *Cryptococcus neoformans*, *Schizosaccharomyces pombe*, *Aspergillus nidulans*, *Yarrowia lipolytica*, CTG-clade yeast *Candida albicans*, protoploid yeast *Lachancea kluyveri*, and post-WGD yeast *Saccharomyces cerevisiae*. The partnerships of characterized and putative homologs involved in three typical types of fungal iron-regulatory systems are shown in the right panel aligned with the phylogenetic tree (rectangles with solid line: characterized genes; rectangles with dash line: uncharacterized genes; blank rectangles with dash line: homolog not identified; green: genes required for iron-uptake regulation; blue: genes required for iron-utilization regulation; rectangles with bold line: genes phenotypically characterized in this study). *Yarrowia lipolytica* was used as an outgroup for *C. albicans* and more recently diverged yeasts. (*B*) Iron-dependent growth of *sef1*Δ and *AFT1*-like factor deletion mutants in *C. albicans*, *L. kluyveri*, and *S. cerevisiae*. *Yarrowia lipolytica* that lacks an Aft1 ortholog was also included for comparison. The mutants showing strong growth defects under iron-deprived conditions have been placed at the bottom of each panel and are highlighted in red. The indicated concentration of the iron chelator BPS (bathophenanthrolinedisulfonic acid disodium salt) was added into YPD plates to create iron-deprived conditions. All plates were incubated at 28 °C for the indicated number of days.

Iron is an essential co-factor in a variety of proteins responsible for DNA synthesis, respiration/electron transport, oxygen transport/storage, and many core metabolic pathways (Drakesmith and Prentice 2008). However, excess intracellular iron can lead to cytotoxicity as a result of the Fenton reaction (Halliwell and Gutteridge 1984). Cells have evolved multiple pathways to maintain homeostasis of intracellular iron concentrations (Canessa and Larrondo 2013; Brault et al. 2015; Bogdan et al. 2016). In extant fungi, three different types of iron regulatory systems have been described (fig. 1*A*). The first type of regulation (Type 1) centers on two transcriptional repressors (HapX-like and GATA factors), which has been identified in *Neurospora crassa*, *Ustilago maydis*, *Cryptococcus neoformans*, *Aspergillus* sp., and *Schizosaccharomyces* sp. (Brault et al. 2015). The second regulatory system (Type 2) works similarly to the Type 1 system but contains an additional transcriptional activator, Sef1, which is essential to induce expression of iron-uptake genes under iron-depleted conditions (Noble 2013). This system has only been characterized in *Candida albicans*, but it is likely conserved in at least the CTG clade *(*containing the species that reassigned the leucine CUG codon to code serine; Santos et al. 2011). The third system (Type 3) was discovered in *Saccharomyces cerevisiae* and it is totally different from Types 1 and 2 (Cyert and Philpott 2013). Instead of transcriptional repressors, it involves the Aft family of transcriptional activators that induce expression of iron-uptake genes and upregulate Cth family proteins that control mRNA degradation of iron-consuming genes (Shakoury-Elizeh et al. 2004; Puig et al. 2005).

As summarized in figure 1*A*, the Type 1 system hypothetically represents an ancient system because it has been identified in both *Basidiomycete* and *Ascomycete* species. Moreover, the absence of *SEF1* and *AFT* family orthologs in the Type 1 species again suggests that the Type 2 and Type 3 systems evolved subsequently. The emergence of *SEF1* in the genome and subsequent incorporation of Sef1 into the Type 1 system probably led to the evolution of the Type 2 system. The Type 3 system could have arisen in two ways: 1) It evolved from the Type 1 system via emergence and incorporation of Aft-family activators and a functional switch of the Type 1 repressors; or 2) it evolved from the Type 2 system with a further functional switch in Sef1 in addition to the events proposed in the first model. Accordingly, we hypothesized that Sef1 plays a central role in the evolution of fungal iron regulatory systems and anticipated that understanding the evolutionary trajectories of Sef1 orthologs in different *Saccharomycotina* species would be a “first” critical step in elucidating the macroevolutionary processes of fungal iron regulatory networks.

To date, comprehensive studies of Sef1 have been limited to *C. albicans*, in which Sef1 directly targets and regulates iron-uptake genes under iron-deprived conditions (Chen et al. 2011). Recently, a study by Gerwien et al. (2016) investigated the function of Sef1 in *S. cerevisiae* and *C. glabrata* and revealed distinct phenotypic outcomes in the respective *sef1*Δ mutants. In *S. cerevisiae*, deletion of *SEF1* did not cause growth defects in iron-depleted conditions but resulted in defects when it was grown in acetate-containing medium. In contrast, deletion of *SEF1* in *C. glabrata* resulted in mild growth defects in both iron-deprived medium and medium containing glycerol as the sole carbon source (but no defects in acetate- or ethanol-containing media). Based on gene expression analyses, Gerwien et al. (2016) postulated that *C. glabrata* possesses a hybrid iron regulatory system consisting of components from both the Type 2 and Type 3 systems. However, ambiguous phenotypic data and a lack of Sef1 ChIP data make it difficult to elaborately characterize the major change in the pathway. Further molecular investigations are required if we want to understand how the Sef1 regulatory network has been rewired. Therefore, we sought a new potential model yeast species exhibiting an explicit switch in function of its Sef1 ortholog relative to *C. albicans* Sef1.

Hence, we investigated the function of Sef1 using various *Saccharomycotina* yeast species that span a nominal 300 My of diversity (Taylor and Berbee 2006). We reveal functional divergence of Sef1 in some of the tested yeast species. We further examined in detail the function of Sef1 in a protoploid yeast (having fewer numbers of chromosomes than the 16-chromosome model species *S. cerevisiae*), *Lachancea kluyveri*, which diverged from the common ancestor of *S. cerevisiae* prior to the yeast whole-genome duplication (WGD) event and occupies a position in the middle of a phylogenetic tree encompassing species spanning from *S. cerevisiae* to *C. albicans* (Génolevures Consortium et al. 2009). Our results indicate that Sef1 in *L. kluyveri* is primarily involved in regulating the tricarboxylic acid (TCA) cycle, with only a minimal contribution to iron uptake. Transcriptional rewiring at the level of regulon membership switching lies behind the functional divergence of Sef1 between *L. kluyveri* and *C. albicans*. Furthermore, we show that a Sef1 gain-of-function *L. kluyveri* mutant with increased transcriptional activity enabled cells to turn on genes unrelated to Sef1’s primary functions. Our data demonstrate a new case of transcriptional rewiring relating to Sef1, implying the presence of a plastically rewired Sef1 transcriptional network encompassing iron-uptake and TCA cycle regulons in the common ancestor of *L. kluyveri* and *C. albicans.* Furthermore, our data suggest that nonfunctional transcription factor (TF) binding may represent a general evolutionary mechanism to increase the potential for gene expression divergence and phenotypic innovation, providing the building blocks for adaptation to environmental perturbations.

## Results

### Sef1 Is Not a Major Contributor to Iron Homeostasis in Tested Non-*C. albicans* Yeast Species, Including *L. kluyveri*

The Sef1 ortholog does not exist outside the *Saccharomycotina* clade (fig. 1*A*, Type 1 species), indicating that Sef1 emerged in the common ancestor of *Saccharomycotina* species. Previous studies have indicated that Sef1 orthologs in *C. albicans*, *S. cerevisiae*, and *C. glabrata* contribute to different regulatory functions (Chen et al. 2011; Noble 2013; Gerwien et al. 2016), suggesting transcriptional rewiring happened at least in the early common ancestor or during the divergence of these species. The subsequent emergence of the Aft factor (fig. 1*A*, Type 2 and 3 species) might facilitate another round of rewiring in the Sef1 network, creating potential interactions between the Aft factor and Sef1. To investigate how Sef1-mediated regulatory networks are altered during yeast evolution, we performed a pilot phenotypic assessment of a diverse group of yeast species—including *S. cerevisiae*, *L. kluyveri*, *Kluyveromyces lactis*, *Hansenula polymorpha*, *Pichia pastoris*, *C. albicans*, and *Yarrowia lipolytica*—to examine their phenotypes in *sef1*Δ backgrounds (fig. 1*B* and supplementary fig. S1*A*, Supplementary Material online). Interestingly, Sef1 was required for growth under iron-deprived conditions solely in *C. albicans*. The protoploid yeasts *L. kluyveri* and *K. lactis* relied on Aft orthologs to grow under low-iron conditions, similar to *S. cerevisiae*, indicating that they generally use the Type 3 iron regulatory system (fig. 1*A* and supplementary fig. S1*A*, Supplementary Material online). In the most distantly related species to this group, *Y. lipolytica*, and in the methylotrophic yeasts *H. polymorpha* and *P. pastoris*, Sef1 orthologs are also not vital for iron-dependent growth. These results suggest that single or multiple functional divergence events have occurred among Sef1 orthologs and also point to the presence of many potential Sef1-centered plastic transcriptional networks across these tested species.

### Unique Genetic Interactions between *SEF1* and *AFT1* in *L. kluyveri* Suggest Crosstalk between the Two Transcriptional Networks

**Fig. 2.**
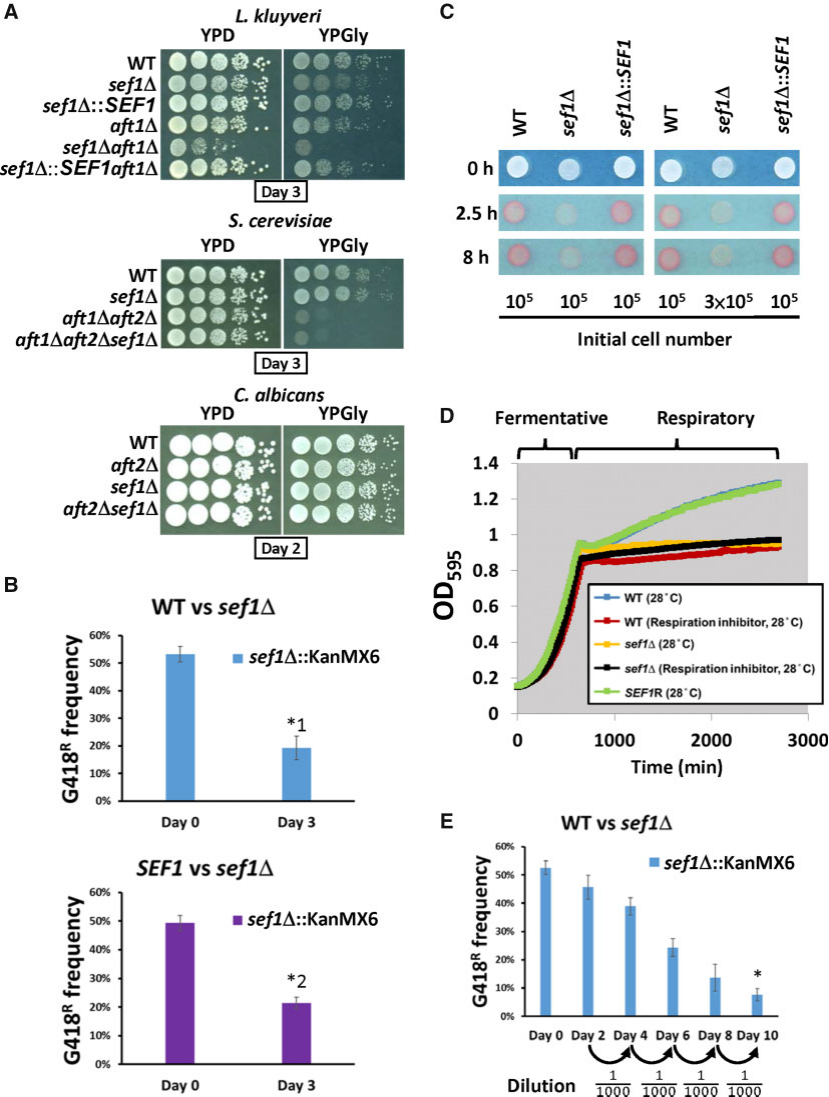
Growth of *Lachancea kluyveri sef1*Δ mutants under fermentative and respiratory conditions. (*A*) The *sef1*Δ mutants exhibit synthetic defects upon deletion mutations of *AFT1* orthologs in *L. kluyveri*, but not in *Saccharomyces cerevisiae* or *Candida albicans*. (*B*) The *L. kluyveri sef1*Δ mutant (G418^R^) is less competitive under normal respiratory conditions. The *sef1*Δ mutant was competed with the wild-type (top panel) or a *SEF1*-reconstituted strain (bottom panel) in YPGly for 3 days at 30 °C with an initial 50:50 cell number ratio. A two-tailed Student’s t-test was used to calculate the *P*-value for each pair by comparing the G418^R^ frequency of the control culture (Day 0) with the G418^R^ frequency of the Day 3 culture. Only the pairs with *P* value < 0.01 are marked with * (*^1^: *P* = 0.000691888; *^2^: *P* = 0.000200426). (*C*) The *L. kluyveri sef1*Δ mutant shows lower respiration-based TTC reduction activities under respiratory conditions (YPGly). The formation of red product in the cell colonies indicates that the cells have competent TTC reduction activity. The whiter spots indicate defects in cellular respiration. (*D*) The *L. kluyveri sef1*Δ mutants show attenuated postdiauxic growth in YPD, similar to the wild type treated with the respiration inhibitor (1 µg/ml antimycin A). The growth curves were measured at 28 °C by using a Tecan plate reader with intermittent shaking. (*E*) The *L. kluyveri sef1*Δ mutant (G418^R^) displays lower competitiveness to the wild type in a bottleneck-based competition assay in YPD. The wild-type and *sef1*Δ mutant competed in YPD with an initial 50:50 cell number ratio for five cycles of 2-day coculture (log- to postdiauxic phase) at 30 °C. A bottleneck was created by subculturing into fresh YPD medium with a 1000-fold dilution between each cycle. A two-tailed Student’s t-test was used to calculate the *P*-value for the final pair by comparing the G418^R^ frequency of the control culture (Day 0) with the G418^R^ frequency of the Day 10 culture. The *P* value marked with * is 1.85104E-05.

In methylotrophic yeasts, deletion of Aft orthologs did not affect iron-dependent growth (supplementary fig. S1*B*, Supplementary Material online). Moreover, our analysis indicated that the *Y. lipolytica* genome does not contain an Aft ortholog. These findings indicate that the Aft factor-mediated iron regulatory pathway probably evolved more recently among yeast species. According to the models described in our Introduction, incorporation of the Aft TF into the iron regulatory network was followed by different levels of plastic rewiring that shaped transcription networks in extant species, such as *C. albicans*, protoploid yeasts, and *S. cerevisiae*. Therefore, we speculated that some evolutionary modifications of the rewired network may still exist in extant species evolutionarily between *C. albicans* and *S. cerevisiae*, such as protoploid yeasts. To test this hypothesis, we assessed synthetic genetic interactions between *SEF1* and *AFT1* in the protoploid yeast *L. kluyveri* to phenotypically evaluate the possibility of network crosstalk. As the *aft1*Δ mutant is excessively sensitive to iron depletion (fig. 1*B*), we examined the fitness of *sef1*Δ*aft1*Δ under iron-rich conditions (without the iron chelator). We observed a clear synthetic defect in the *sef1*Δ*aft1*Δ double mutant of *L. kluyveri* compared with single deletion strains (fig. 2*A*). Given that perturbations of iron homeostasis interfere with iron-dependent metabolic pathways (i.e., those involving proteins with iron as a cofactor or structural component), such as respiration and the TCA cycle (Philpott et al. 2012), we also confirmed the appearance of a more severe synthetic effect in double mutants grown in a nonfermentable carbon source (YPGly) in which functional respiration is required. In contrast, *sef1*Δ*aft1*Δ double mutants of *S. cerevisiae* and *C. albicans* grew similarly to single mutants and wild-type cells (fig. 2*A*). The unique genetic interaction between *SEF1* and *AFT1* in *L. kluyveri* not only indicates that *L. kluyveri*-Sef1 (denoted Lk-Sef1) functions differently from *S. cerevisiae*-Sef1 (denoted Sc-Sef1) and *C. albicans*-Sef1 (denoted Ca-Sef1) but also implies that there is crosstalk between the Lk-Sef1 and Lk-Aft1 networks, likely representing a hybrid feature of plastic rewiring patterns when the species diverged from its common ancestor.

### Sef1 in *L. kluyveri* Is Required for Full Growth under Respiratory Conditions

When we characterized the phenotypes of *sef1*Δ mutants in different species, we found that *Lk*-*sef1*Δ cells, but not *Sc*-*sef1*Δ or *Ca*-*sef1*Δ cells, exhibited mild growth defects in a nonfermentable carbon source (YPGly) (fig. 2*A* and *B*). To assess their efficiency in cellular respiration, we performed a triphenyl tetrazolium chloride (TTC) reduction assay. The *Lk*-*sef1*Δ mutant indeed displayed weaker TTC reduction activity (white color) relative to the wild-type and reconstituted strain (red color, fig. 2*C*). It is well known that heat stress increases cellular demands of ATP from respiration (Lahtvee et al. 2016) and that competent respiration is required for cells to activate stress responses at high temperatures (Zubko and Zubko 2014). Consistently, we observed that *Lk*-*sef1*Δ cells exhibited more severe growth defects at 39 _**°**_C (supplementary fig. S2*A*, Supplementary Material online). Moreover, deletion of *Lk*-*SEF1* significantly reduced postdiauxic growth, resulting in a growth curve similar to that generated for respiration-inhibited wild-type cells (fig. 2*D*). When *Lk*-*sef1*Δ and wild-type cells were subjected to a cycled competition assay that included the postdiauxic growth phase, we observed that the *sef1*Δ mutant was less competitive (fig. 2*E*).

Together, these data reveal that Lk-Sef1 plays a role in respiratory growth. We also examined whether Sef1 contributed to respiratory growth in *K. lactis*, another protoploid yeast, and *Y. lipolytica*, representing an outgroup species for *C. albicans* and more recently diverged species. Interestingly, Kl-Sef1 was not required for respiratory growth, and the deletion mutant gained slight heat resistance (supplementary fig. S2*B*, Supplementary Material online). Deletion of *SEF1* from *Y. lipolytica* did not result in any phenotype under either condition (supplementary fig. S2*C*, Supplementary Material online). The phenotypic outcomes for *sef1*Δ mutants of different species support the hypothesis that multiple functional switch events have occurred among Sef1 orthologs across these tested species (supplementary fig. S2*D*, Supplementary Material online).

### Sef1 in *L. kluyveri* Is a Transcriptional Activator Induced by Nonfermentable Carbon Sources

**Fig. 3.**
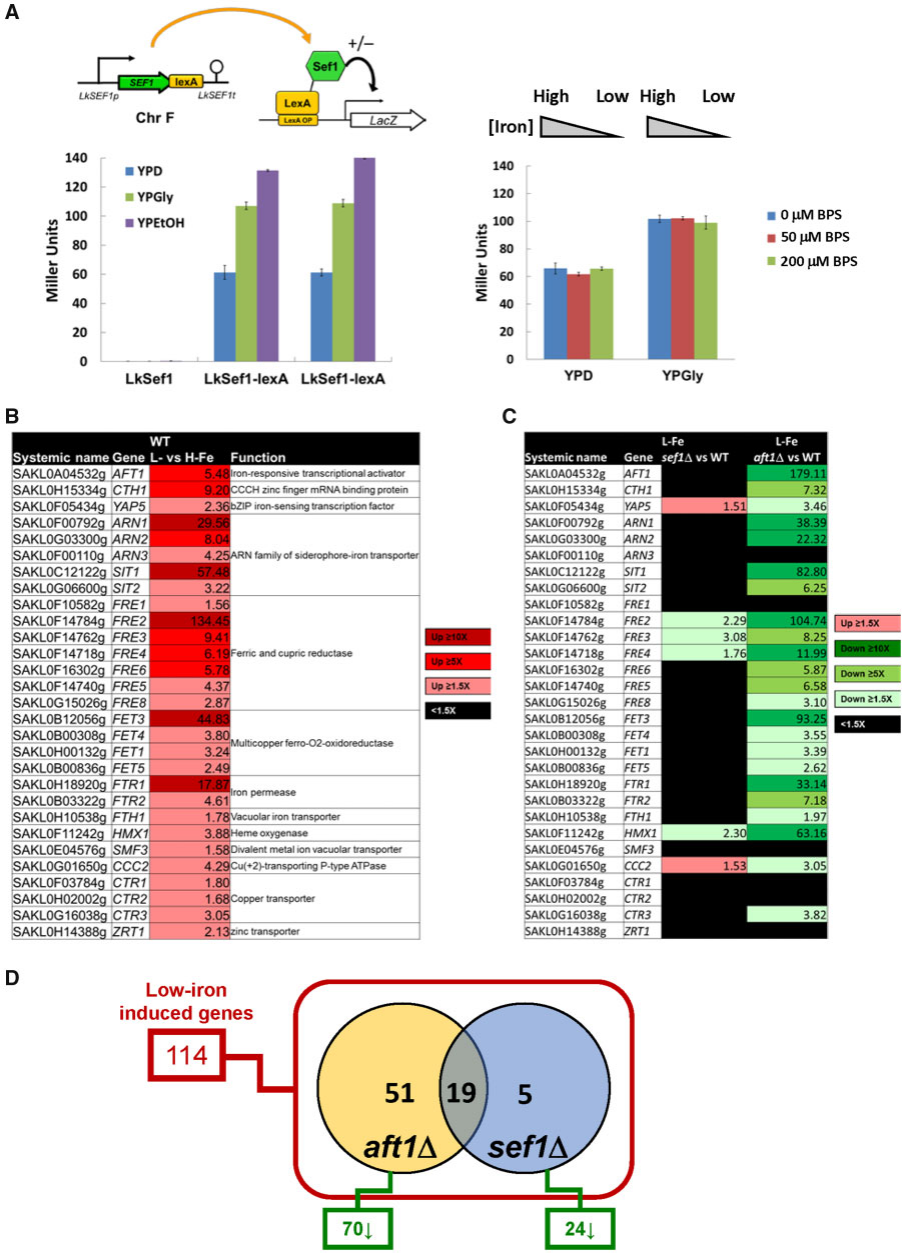
Sef1-mediated iron-dependent gene expression analyses in *Lachancea kluyveri*. (*A*) One-hybrid assays for *L. kluyveri* Sef1. Lk-Sef1 activates transcription in response to both fermentative (glucose) and respiratory (glycerol and ethanol) carbon sources (left panel), but not to environmental iron levels (right panel). The native-promoter-driven Sef1, which is C-terminally fused with the LexA DNA-binding domain, binds to the LexA operator (lexAOP) on the basal promoter of the *lacZ* reporter gene and modulates the expression of *lacZ*. LacZ activity was measured using the liquid-galactosidase assay. In the left panel, two independent clones of the Sef1-lexA one-hybrid strain were assayed. The β-galactosidase levels are displayed as average Miller units ± SD from at least three technical repeats. Because this system was constructed on the native *SEF1* locus, the nontagging (LexA-less) strain was used as a negative control. The LexA DNA-binding domain-only had no artificial effect on transcription (see fig. 5*C*). (*B*) A total of 29 low-iron-induced genes identified as being potentially involved in iron uptake in wild-type *L. kluyveri.* (*C*) The dependency of low-iron-induced iron-uptake-related genes on Sef1 or Aft1 in *L. kluyveri* is shown. Gene expression of the mutants was compared with the wild type. Upregulated genes in each mutant with ≥1.5-fold change are highlighted in red. Downregulated genes in each mutant with ≥1.5-fold change are highlighted in green. Nondifferentially expressed genes (<1.5-fold change) are marked in black. (*D*) The dependency of low-iron-induced genes on Sef1 and/or Aft1 in *L. kluyveri* is displayed in the Venn diagram. Of the 114 low-iron-induced genes defined in wild type, 70 and 24 genes are downregulated in the *aft1*Δ and *sef1*Δ mutants, respectively, in comparison to wild type. For (*B*), (*C*), and (*D*), the RNA expression profiles from the *L. kluyveri* wild-type, *sef1*Δ, and *aft1*Δ cells grown to mid-log-phase under both high-iron (YPD + 400 µM ferrous ammonium sulfate) and low-iron (YPD + 200 µM BPS) conditions were compared. At least 3–4 biological repeats of each sample were analyzed. The details of analyses are shown in Materials and Methods.

Expression or activity of TFs often changes in response to specific conditions. To investigate whether Lk-Sef1 is subject to such regulation, we generated Lk-Sef1-lexA fusion protein constructs and performed lexA-dependent one-hybrid assays under different conditions (fig. 3*A*). Lk-Sef1 behaved like a typical transcriptional activator that could turn on the downstream *LacZ* reporter gene in the glucose medium. Moreover, the activity of Lk-Sef1 was enhanced when cells grew in nonfermentable carbon sources, but not in an iron-depleted medium. This result corroborates our *sef1*Δ mutant data that the Lk-Sef1-mediated transcriptional network is likely involved in respiratory growth.

To further investigate the crosstalk between Sef1 and Aft1, we examined the transcriptome profiles of *L. kluyveri sef1*Δ, *aft1*Δ, and wild-type cells (supplementary tables S1 and S2, Supplementary Material online) under normal and low-iron conditions. We first confirmed our test conditions by defining and characterizing the induction of 29 iron-uptake-related genes in wild-type cells under the low-iron condition (fig. 3*B*). As both Sef1 and Aft1 are transcriptional activators, we speculated that their mutation may affect the expression of the same set of iron-uptake genes. In the *aft1*Δ mutant, expression of most of the iron-uptake-related genes (23/29) was reduced compared with wild-type cells. In contrast, the expression of only four genes (4/29) was significantly reduced in *sef1*Δ cells (fig. 3*C*). Including these iron-uptake-related genes, a total of 114 genes was upregulated in wild-type cells under low-iron conditions. Among these 114 genes, anticipated upregulation was hindered in 24 and 70 of them in the *sef1*Δ and *aft1*Δ lines, respectively. Interestingly, 19 out of the 24 (79%) genes affected in the *sef1*Δ line were also affected in the *aft1*Δ line (fig. 3*D* and supplementary table S2, Supplementary Material online), suggesting that Lk-Sef1 and Lk-Aft1 genetically share many downstream targets in iron-related pathways. These findings support our phenotypic results that Lk-Sef1 only plays a minor role in iron homeostasis and that a plastic rewiring of the Sef1 transcriptional network shifted its biological functions to regulate other phenotype-determining genes.

### TCA Cycle-Related Genes Are the Major Binding Targets of Lk-Sef1

Our results indicate that the function of Lk-Sef1 has deviated from that of Sef1 in *C. albicans*, probably via plastic rewiring onto its downstream target genes upon divergence from its common ancestor. To directly establish the identity of those target genes, we performed chromatin immunoprecipitation-sequencing (ChIP-seq) experiments on Lk-Sef1 from cells grown under YPD or YPGly conditions. A total of 895 consistent peaks were identified across all three biological repeats in the same condition, including 378 peaks called from YPD samples and 780 peaks called from YPGly samples (supplementary fig. S3*A* and table S3, Supplementary Material online). Both numbers of peaks and enrichment scores were higher in YPGly than in YPD (supplementary fig. S3*B*, Supplementary Material online), consistent with the phenotypic characterization showing that Lk-Sef1 has a greater influence on respiratory growth.

**Fig. 4.**
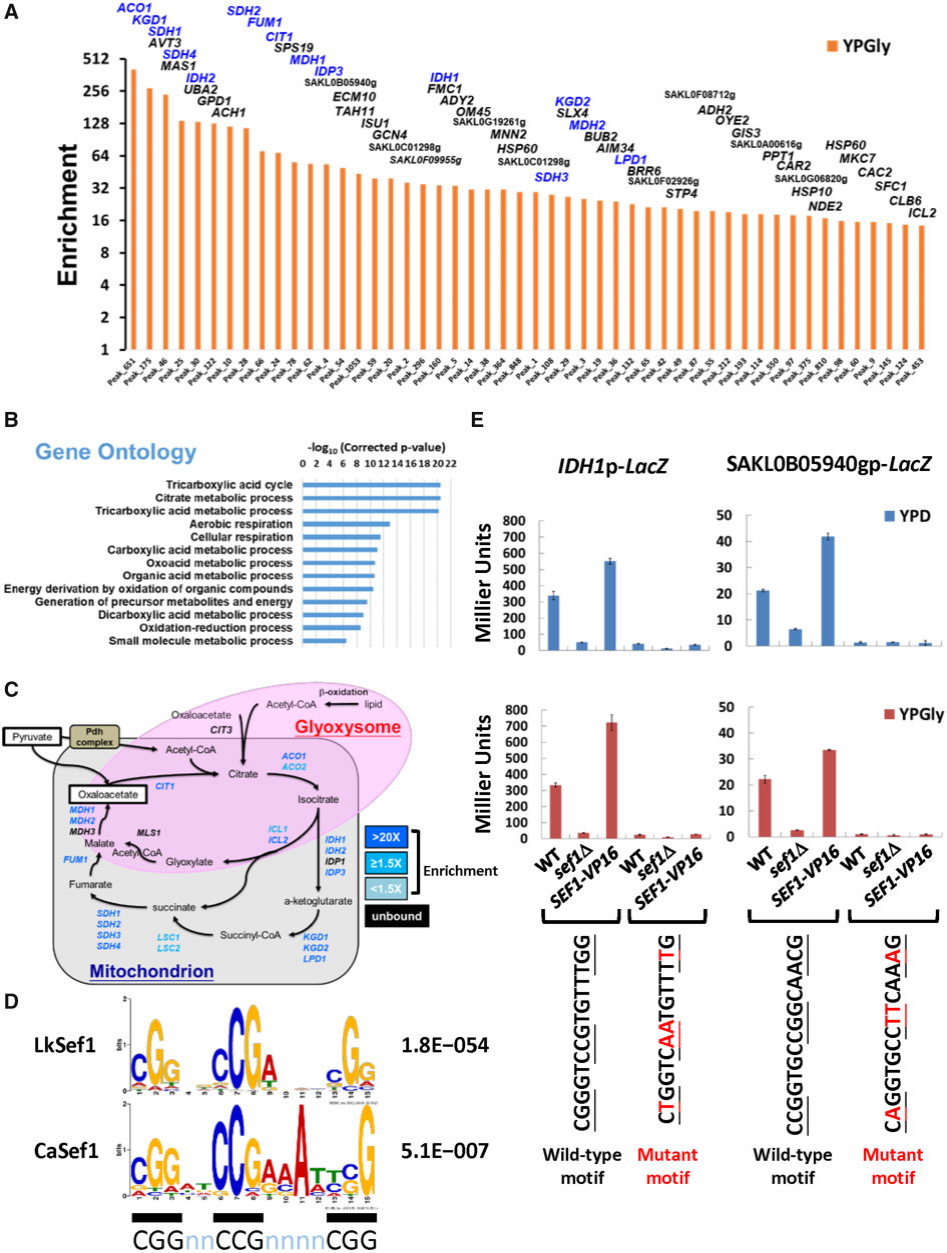
Lk-Sef1 direct targets are strongly enriched in TCA cycle genes. (*A*) TOP50 peaks ranked based on the enrichment values under YPGly conditions. All 895 peaks (Peak_N, *x*-axis) were ranked based on ChIP enrichment values (*y*-axis) in descending order (orange bars) (also see supplementary fig. S3, Supplementary Material online, and associated figure legend) and only the TOP50 peaks are shown. The names of target genes assigned for each peak are labeled on top of each bar, with TCA cycle genes highlighted in blue. (*B*) GO analysis for target genes from the TOP50 peaks. There are 56 *Lachancea kluyveri* target genes assigned to TOP50 peaks, which corresponded to 51 *Saccharomyces cerevisiae* orthologs. GO information borrowed from *S. cerevisiae* (SGD; GO Term Finder, version 0.86) was used to analyze the 51 orthologs. The enriched GO terms with a corrected *P*-value lower than 0.0001 are shown. (*C*) A schematic diagram of the TCA cycle and glyoxylate shunt. Both genes and pathways are included. Genes targeted by *L. kluyveri* Sef1 with enrichment values >1.5-fold in either YPD or YPGly conditions are highlighted in blue. Genes that are not targeted by Sef1 are marked in black. (D) The *L. kluyveri* Sef1 DNA recognition motif compared with the published consensus binding motif of *Candida albicans* Sef1. MEME-ChIP was used to generate a position weight matrix (PWM) file of *the L. kluyveri* Sef1 DNA recognition motif. The 250-bp sequences flanking each peak center of the TOP450 peaks were used in analyses. The *C. albicans* Sef1 motif was re-created by repeating the MEME analysis based on previous work (Chen et al. 2011). The consensus “CGGnnCCGnnnnCGG” motif of PWM logos is underlined with black bars. The accompanying *E*-value for each de novo motif prediction is indicated. (*E*) Verification of Sef1 motif prediction by the LacZ reporter-based promoter assay. The peak regions for the *L. kluyveri IDH1* and SAKL0B05940g promoters contain only one most high-confidence (*P*-value < 0.0001) Sef1 binding motif. The wild-type and motif-mutated promoters were fused to the LacZ reporter gene. The mutated residues on each motif are highlighted in red. The contribution of Sef1 motifs to promoter activities was assayed in *L. kluyveri* wild-type, *sef1*Δ, and highly active *SEF1-V16* cells. The LacZ activity was measured under both YPD and YPGly conditions using the liquid-galactosidase assay. The β-galactosidase levels are displayed as average Miller units ± SD from at least three technical repeats.

Next, we analyzed the top 50 most strongly enriched peaks from YPGly samples. Gene ontology (GO) analysis revealed that the TCA cycle and respiration genes were the most enriched (fig. 4*A* and *B*). Moreover, 15 out of 21 TCA cycle genes annotated in the *L. kluyveri* genome were in the top-50 list (fig. 4*C*). A subsequent survey of the 895 total peaks (corresponding to 758 assigned genes) revealed that 18 TCA cycle genes were bound by the TF Lk-Sef1 (fig. 4*C* and supplementary fig. S4, Supplementary Material online). In contrast, only four out of 29 iron-uptake-related genes were bound by Lk-Sef1. These results indicate that TCA-cycle-related genes are the major binding targets of Lk-Sef1.

To further examine Sef1-mediated transcriptional regulation, we generated Lk-Sef1-binding motifs de novo using the sequences of the top-450 Sef1-binding peaks. We selected one of the motif candidates that was highly similar to the known Sef1-binding motif from *C. albicans* (Chen et al. 2011) and that possessed the consensus feature of DNA recognition motifs of the zinc cluster protein family (MacPherson et al. 2006) (fig. 4*D*). A subsequent motif scan demonstrated that all 895 peak regions contained putative binding motifs (FIMO *P*-value < 0.05, representing the probability of a random sequence of the same length as the motif matching that position of the sequence with a score at least as good as the motif; supplementary fig. S4, Supplementary Material online). Moreover, high-confidence motifs (*P*-value < 0.0001) were observed in the promoters of the 15 TCA cycle genes strongly bound by Lk-Sef1 (supplementary fig. S4, Supplementary Material online).

Our identification of this binding motif allowed us to investigate directly the effect of Lk-Sef1 binding on gene expression. We chose two promoters carrying high-confidence motifs from the top-50 Sef1-binding list (including one TCA cycle gene *IDH1* and one uncharacterized gene SAKL0B05940g) and generated corresponding plasmid-based LacZ reporter constructs (supplementary fig. S5*A*, Supplementary Material online). By introducing mutations into the conserved residues of the Sef1-binding motif (supplementary fig. S5*B*, Supplementary Material online), we were able to completely abolish reporter expression (fig. 4*E*). These results confirm that the de novo-predicted Lk-Sef1-binding motif is functional and contributes to Lk-Sef1-mediated gene expression.

### Pervasive Nonfunctional Binding of Sef1 on Its Target Genes

**Fig. 5.**
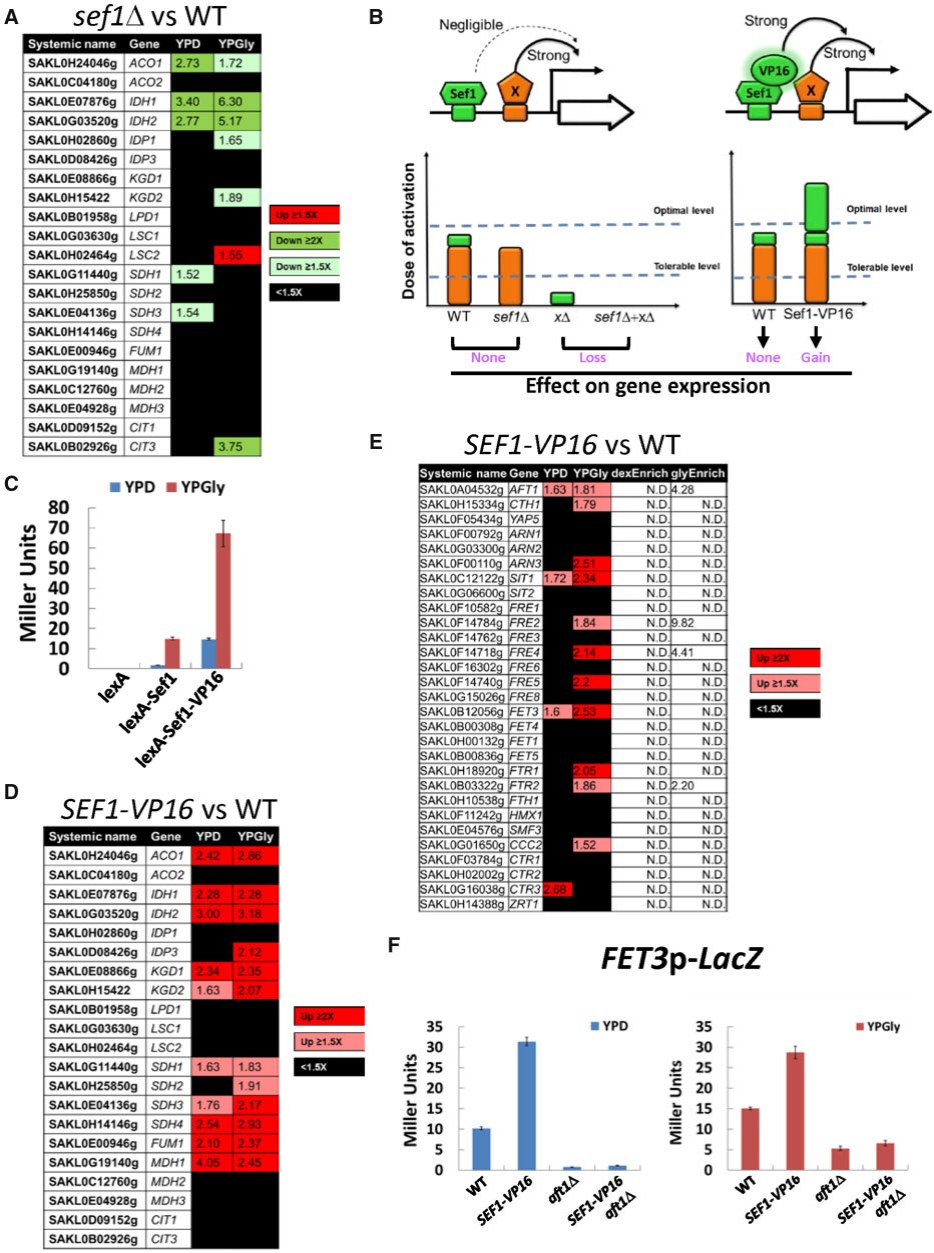
The contribution of *Lachancea kluyveri* wild-type Sef1 and the highly active Sef1 mutant to the expression of direct target genes. (*A*) Differential expression of TCA cycle genes in response to *sef1*Δ. The RNA expression profiles from the *L. kluyveri* wild-type and *sef1*Δ cells grown to early log-phase under both YPD and YPGly conditions were compared. A total of 21 annotated TCA cycle genes are listed and 18 of them are direct targets of Sef1 with three exceptions, *IDP1*, *MDH3*, and *CIT3*. (*B*) Proposed model of “insufficient dose of activation” on target gene expression for *L. kluyveri* Sef1. Left panel: The activation activity of Sef1 is negligible relative to strong transcription factor X. Therefore, deletion of *SEF1* does not affect gene expression. Right panel: Sef1 transcriptional activity is enhanced by fusing with a VP16 domain. When the additive activation dose from Sef1-VP16 and factor X exceeds the threshold of the optimal level, gene expression will be further induced. “Tolerable level” represents the minimal dose of transcriptional activation to maintain the wild-type gene expression level. “Optimal level” is the minimal dose of transcriptional activation to gain higher gene expression than the wild-type level. (*C*) One-hybrid assays for C-terminally VP16-fused *L. kluyveri* Sef1. The Sc-ADH1 promoter-driven Lk-Sef1 and Lk-Sef1-VP16, which are N-terminally fused with LexA protein, bind to the LexA operator (lexAOP) on the basal promoter of the *lacZ* reporter gene and modulate the expression of *lacZ*. LacZ activity was measured using the liquid-galactosidase assay. The β-galactosidase levels are displayed as average Miller units ± SD from at least three technical repeats. (*D*) Differential expression of TCA cycle genes in response to the gain-of-function *SEF1-VP16* mutation. The RNA expression profiles from the *L. kluyveri* wild-type and *SEF1-VP16* cells grown to early log-phase under both YPD and YPGly conditions were compared. (*E*) Differential expression of iron-uptake-related genes in response to the gain-of-function *SEF1-VP16* mutation. The RNA expression profiles from the *L. kluyveri* wild-type and *SEF1-VP16* cells grown to early log-phase under both YPD and YPGly conditions were compared. The terms “dexEnrich” and “glyEnrich” represent enrichment values of Sef1-binding from ChIP-seq data under glucose and glycerol conditions, respectively. “N.D.” means the gene is not targeted by Sef1. (*F*) The activities of the *FET3* promoter were assayed in *L. kluyveri* wild-type, *SEF1-V16*, *aft1*Δ, and *SEF1-VP16*/*aft1*Δ cells. LacZ activity was measured under both YPD and YPGly conditions using the liquid-galactosidase assay. The β-galactosidase levels are displayed as average Miller units ± SD from at least three technical repeats. For (*A*), (*D*), and (*E*), upregulated genes in each mutant with ≥1.5-fold change are highlighted in red. Downregulated genes in each mutant with ≥1.5-fold change are highlighted in green. Nondifferentially expressed genes (<1.5-fold change) are marked in black. At least 3–4 biological repeats of each sample were analyzed. The details of analyses are shown in Materials and Methods.

The strong enrichment for Sef1 binding to TCA cycle genes would seem to conflict with the mild respiratory growth defects observed in *sef1*Δ cells (fig. 2*A*). This scenario raises the question of whether the expression of TCA cycle genes is regulated by Sef1. Indeed, when we compared the transcriptome profiles of *sef1*Δ and wild-type cells (supplementary tables S4 and S5, Supplementary Material online), only 16 and 96 genes were respectively downregulated under YPD and YPGly conditions in the *sef1*Δ mutant, including only eight TCA cycle genes (fig. 5*A*). We used qPCR to further confirm that some of the Sef1-binding TCA cycle genes are insensitive to *sef1*Δ (supplementary fig. S6, Supplementary Material online). Consequently, for those insensitive genes, Lk-Sef1 acts like a “spurious” regulator, binding strongly to the target genes but not contributing to their expression.

To verify whether the growth defect of *Lk-sef1*Δ cells under respiratory conditions is caused by downregulation of the Sef1-bound TCA cycle genes, we chose one of them, *ACO1*, which has the most abundant mRNA level among Sef1-regulated TCA cycle genes, and overexpressed it via a strong promoter (from SAKL0B08294g encoding the translational elongation factor Tef2). Sole overexpression of the *ACO1* gene partially restored the fitness of *Lk-sef1*Δ cells (supplementary fig. S7, Supplementary Material online), indicating that the TCA cycle genes are indeed the major phenotype-determining genes regulated by Sef1 under respiratory conditions. Furthermore, only a small proportion of Sef1-bound genes was downregulated in response to *sef1*Δ, representing 3.96% (30/758) of all Sef1 targets (compare supplementary tables S3, S4, and S5, Supplementary Material online). These results imply that the majority of Lk-Sef1-binding genes are subjected to more complex regulation, possibly through other redundant or additive transcriptional regulators.

### Enhanced Sef1 Transcriptional Activity in *L. kluyveri* Alters the Native Transcriptional Network

Nonfunctional TF binding (“spurious” TF binding) describes the binding of a TF on the promoter of a target gene that serves no function in driving gene expression (Spivakov 2014). The thresholds of dose-of-activation on the promoter determine functional redundancy of each bound TF for gene expression (fig. 5*B*) (Spivakov 2014). As Lk-Sef1 binds to 895 regions across the genome, spanning 758 genes with different binding affinities (supplementary table S3, Supplementary Material online), extrinsic (environmental) or intrinsic (genetic) perturbations of Sef1 may change the gene expression pattern of this transcriptional network and consequently alter phenotypic diversity. One possible perturbation is to vary its potency or redundancy in regulating target genes so that nonfunctional binding can become functional. To test this hypothesis, we fused Lk-Sef1 with the activation domain of a viral protein, VP16, known to interact with the TATA-binding protein, TFIIB, and the SAGA histone acetylase complex to enhance yeast transcription (Hall and Struhl 2002; Hirai et al. 2010; Stynen et al. 2012), and then examined its effects on known Sef1-binding targets (fig. 5*B*). Addition of the VP16 activation domain (hereafter, named VP16) indeed increased the basal activation activity of Lk-Sef1 (fig. 5*C*), as well as the expression of tested Sef1-binding targets (supplementary fig. S8*A*, Supplementary Material online). When the transcriptome profiles of *SEF1-VP16* and wild-type cells were compared, 85 and 92 genes were, respectively, upregulated under YPD or YPGly conditions (supplementary tables S6 and S7, Supplementary Material online), including six additional TCA cycle genes previously known to be insensitive to *sef1*Δ (fig. 5*D*). These results demonstrate that by enhancing the transcriptional activity of a TF to mimic its acquisition of gain-of-function mutations, nonfunctional TF binding can partially and selectively (i.e., conditionally) become functional, allowing the TF to gain control of some target genes originally unaffected by it. We have defined this phenomenon as “resuscitation” in this study.

### A Resuscitated Lk-Sef1 Transcriptional Network Endows Immediate Adaptive Benefits in Changing Environments

Next, we wondered whether a resuscitated transcriptional network could endow cells with immediate adaptive potential. To do this, we examined the phenotypic outcomes of the resuscitated Sef1-VP16 transcriptional network. Interestingly, we found that 12 iron-uptake-related genes were upregulated in *SEF1-VP16* cells (fig. 5*E*), despite Sef1 binding being enriched in only four of these 12 iron-uptake genes (i.e., *AFT1*, *FRE2*, *FRE4*, and *FTR2*, see supplementary table S3, Supplementary Material online). We speculated that Sef1-VP16 activated expression of those iron-uptake-related genes indirectly by increasing *AFT1* levels given that TF *AFT1* is one of the Sef1-binding targets (supplementary fig. S4, Supplementary Material online). We constructed a *FET3* promoter-*LacZ* reporter to test this hypothesis. We found that *FET3* expression was upregulated in the *SEF1-VP16* background, but deletion of *AFT1* abolished that effect (fig. 5*F*). Using qPCR, we further confirmed these Aft1-dependent effects in another two non-Sef1-bound iron-uptake genes (*SIT1* and *FTR1*) (supplementary fig. S8*B*, Supplementary Material online). These results reveal an additional layer of crosstalk between the Lk-Sef1 and Lk-Aft1 networks at the molecular level.

**Fig. 6.**
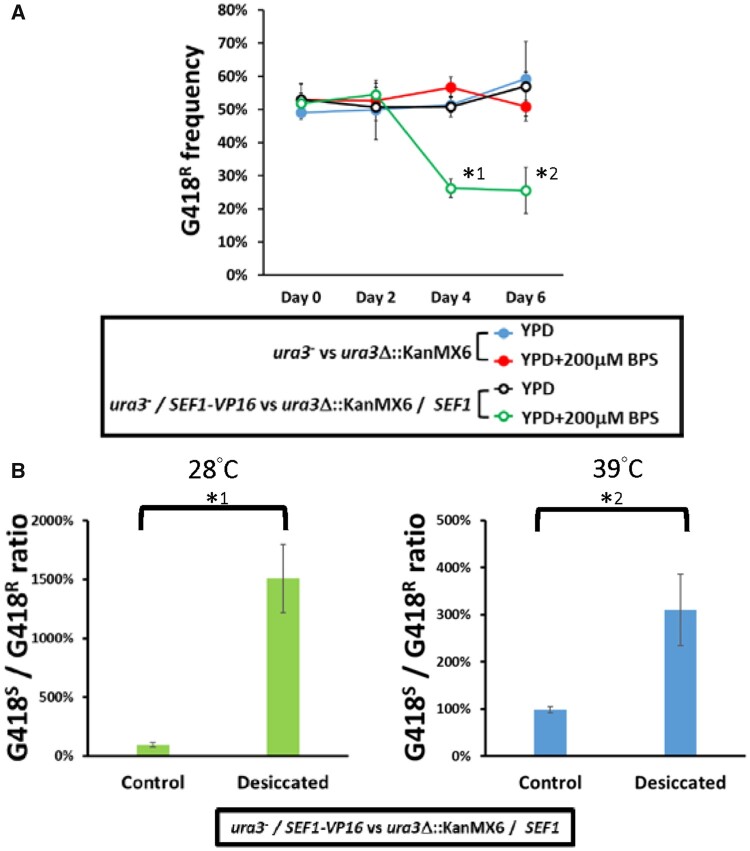
Fitness consequences of the highly active Sef1 strain in *Lachancea kluyveri*. (*A*) The *SEF1-VP16* mutant in *L. kluyveri* is more competitive than the wild type under low-iron conditions. Competition assays were performed by mixing the tested strains (*ura3*^−^; G418-sensitive) with the reference wild-type strain (*ura3*Δ::KanMX6; G418-resistant) at an initial 50:50 cell number ratio for 6 days at 30 °C. Four sets of the competition were conducted as follows: (blue closed circle) the G418-sensitive wild type was competed with the G418-resistant wild type in YPD; (red closed circle) the G418-sensitive wild type was competed with the G418-resistant wild type in YPD + 200 µM BPS; (black open circle) the G418-sensitive *SEF1-VP16* mutant was competed with the G418-resistant wild type in YPD; and (green open circle) the G418-sensitive *SEF1-VP16* mutant was competed with the G418-resistant wild type in YPD + 200 µM BPS. Each frequency of G418-resistant cells is shown on the *y*-axis at the indicated time (2-day intervals). A two-tailed Student’s t-test was used to calculate the *P*-value for each pair by comparing the frequencies at Day 0 with the frequencies for the other days. Only the pairs with *P* value <0.01 are marked with * (*^1^: *P* = 9.09665E − 07; *^2^: *P* = 0.00036). (*B*) The *SEF1-VP16* mutant in *L. kluyveri* is more resistant to desiccation than the wild type. Desiccation assays were performed by mixing the *SEF1-VP16* strain (*ura3*^−^; G418-sensitive) with the reference wild-type strain (*ura3*Δ::KanMX6; G418-resistant) at an initial 50:50 cell number ratio and air-dried for 24 h at 28 or 39 °C. Each ratio of G418-sensitive to -resistant cells is shown. A two-tailed Student’s t-test was used to calculate the *P*-value for each pair by comparing the G418^S-to-R^ ratio of the control sample (without desiccation) with the G418^S-to-R^ ratio of the desiccated sample. Only the pairs with *P* value <0.01 are marked with * (*^1^: *P* = 0.004867043; *^2^: *P* = 0.000600783).

The fact that iron-uptake-related genes were upregulated in the *SEF1*-*VP16* strain implies that this scenario may benefit cells in an environment with fluctuating iron availability. We tested this idea by a competition assay between wild-type and *SEF1-VP16* cells grown under iron-rich or low-iron conditions. Our results show that the *SEF1-VP16* strain grew equally as well as the wild-type strain under iron-rich conditions but outcompeted the wild-type cells when iron was depleted (fig. 6*A*). Previously, the TCA cycle was shown to be involved in the desiccation tolerance of worms and yeast (Erkut et al. 2016). We found that the *SEF1-VP16* strain also exhibited higher desiccation tolerance than the wild-type strain (fig. 6*B*), with _**∼**_3-fold improved tolerance when desiccated at 28 _**°**_C and a 15-fold improvement at 39 _**°**_C. Together, our findings provide evidence that rendering nonfunctional TF binding on a transcriptional network functional via newly acquired regulatory mutations enables a cell to better adapt to a changing environment. If such conditions persist, then the newly acquired regulatory function may be further adjusted, leading to subsequent transcriptional network rewiring.

## Discussion

### Plastic Rewiring of Sef1 Transcriptional Networks across Species

**Fig. 7.**
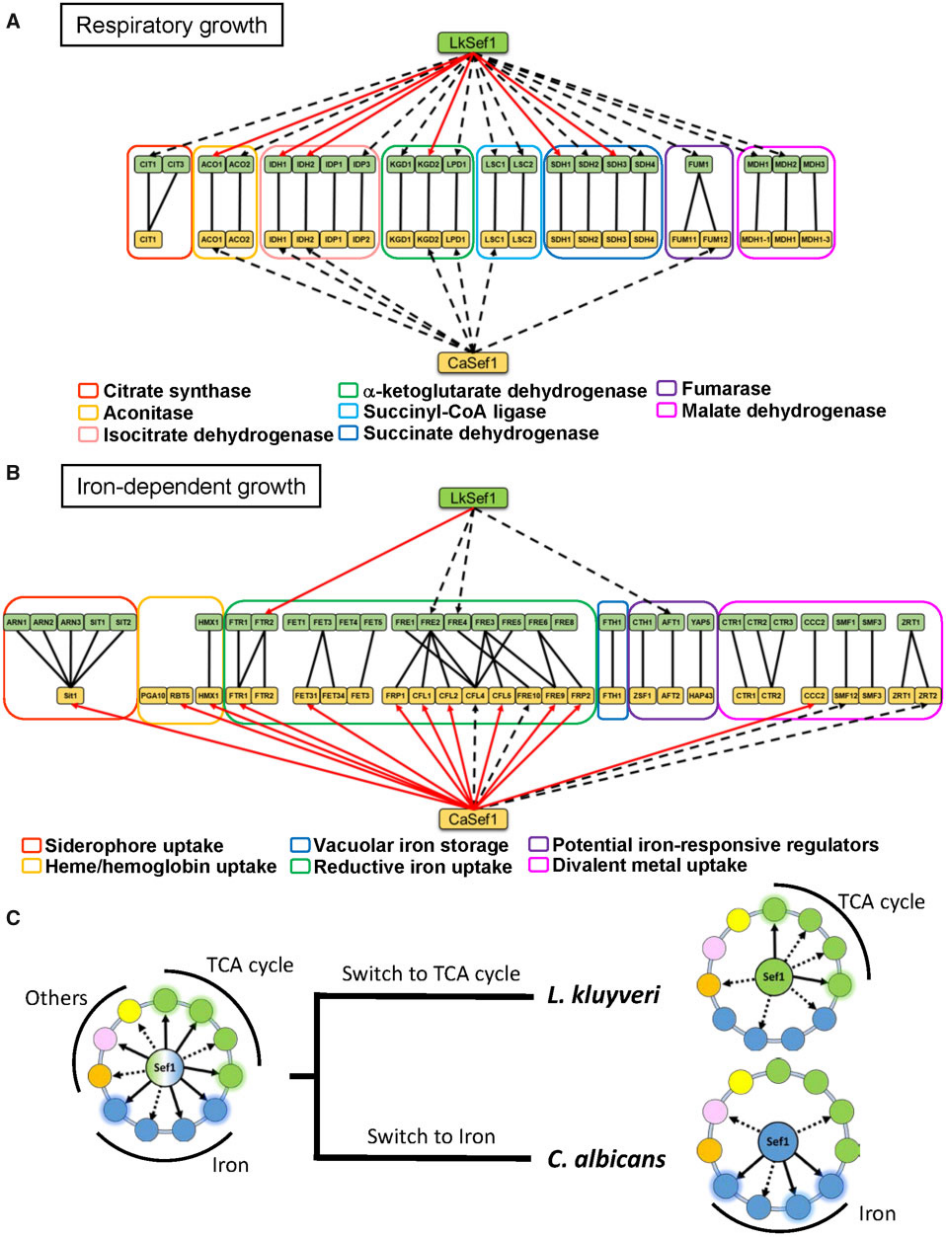
Summary of Sef1 plastic rewiring by comparing Lk-Sef1 with Ca-Sef1. (*A*) Direct TCA cycle gene regulation by Sef1 in *Lachancea kluyveri* and *Candida albicans.* (*B*) Direct iron-uptake-related gene regulation by Sef1 in *L. kluyveri* and *C. albicans.* The dashed black arrows indicate Sef1 binding to the promoter of the target gene, as confirmed by ChIP-seq data. The solid red arrows represent that Sef1 not only targets the gene directly but also is essential for gene expression according to microarray analyses. The homologous groups of TCA cycle genes or iron-uptake-related genes are classified by differently colored rectangles as indicated. (*C*) The proposed switches in the function of Sef1 from a hypothetical common ancestor.

Deletion of *SEF1* orthologs from diverse yeast species resulted in them displaying a variety of mutant phenotypes (figs. 1 and 2 and supplementary fig. S1, Supplementary Material online) (Gerwien et al. 2016), suggesting that the function of Sef1 has changed multiple times during the evolution of *Saccharomycotina* yeasts. The high plasticity of Sef1-mediated regulation is best exemplified by the iron-uptake regulatory pathway, in which Sef1 is a master regulator in *C. albicans* but plays no role in *S. cerevisiae* (Shakoury-Elizeh et al. 2004; Philpott et al. 2012; Noble 2013). By investigating Sef1 in a protoploid yeast species, *L. kluyveri*, we have revealed a functional divergence of Sef1 between two crosstalking pathways, that is, iron uptake and the TCA cycle. Although both Lk-Sef1 and Ca-Sef1 bind multiple targets in the iron-uptake and TCA cycle pathways, there has been a significant reciprocal reduction in the numbers of binding targets in both pathways (fig. 7*A* and *B*). The Lk-Sef1 network has more connections to the TCA cycle pathway, whereas Ca-Sef1 plays a more significant role in regulating the iron-uptake pathway. Moreover, the previous *sef1*Δ transcriptome data also indicated that Sef1 orthologs affect the expression of three TCA cycle genes (*ACO1*, *IDH1*, and *IDH2*) and a small set of iron-uptake genes in *S. cerevisiae* (Reimand et al. 2010; Kemmeren et al. 2014) and *C. glabrata* (Gerwien et al. 2016), although whether this regulation is direct or indirect is unknown because there are no corresponding ChIP data and the precise binding targets of Sc-Sef1 and Cg-Sef1 have not been identified. The observed plastic Sef1-mediated regulation of TCA cycle genes and iron-uptake genes in species from different phylogenetic clades implies that Sef1 possessed both TCA cycle and iron-uptake genes as binding targets in the common ancestor of those species. The extensive prevalence of the Lk-Sef1 motif (fig. 4*D*) on the TCA cycle genes of other *Lachancea* species supports this idea (supplementary fig. S9, Supplementary Material online), suggesting that *L. kluyveri* may not be the unique *Lachancea* species using Sef1 to regulate TCA cycle genes. We propose a simplified model showing that both plastic rewiring of transcriptional networks and different functional switches of Sef1 led to the current Sef1 networks in *L. kluyveri* and *C. albicans* lineages (fig. 7*C*). However, it remains unclear whether the vestigial regulation of Aft1 by Sef1 is derived from the common ancestor or a lineage-specific evolution in *L. kluyveri*. Nevertheless, the functional divergence of Aft orthologs between *C. albicans* and *L. kluyveri* implies that there is another transcriptional rewiring among Aft factors. It will be interesting to investigate how these two rewired regulatory networks (Aft and Sef1) are coupled or have diverged during evolution.

### Potential Adaptive Roles of Nonfunctional Sef1-Binding Events

For many well-studied TFs, the numbers of ChIP-identified target genes are much higher overall than for the numbers of verified TF-regulated genes (Chen et al. 2011; Lane et al. 2015; Merhej et al. 2016; Kuang et al. 2017; Mittal et al. 2017; Pincus et al. 2018; Ronsmans et al. 2019). If we solely consider the TFs that can bind to more than 50 target genes (>1% of total genes in the genome), their functional binding frequencies (i.e., the number of genes bound and regulated by the TF/the number of genes bound by the TF) vary from 6.8% (Merhej et al. 2016) to 87.9% (Mittal et al. 2017). Interestingly, comparing with Ca-Sef1, which has a 50% functional binding frequency (Chen et al. 2011), Lk-Sef1 has an even lower functional binding frequency (3.96%) (supplementary tables S3–S5, Supplementary Material online), highlighting the relatively pervasive nonfunctional binding of Sef1 on its target genes. These nonfunctional binding targets in the Sef1 network probably result from plastic rewiring, but they may also serve as building blocks for the formation of a network with new biological functions. This idea is supported by the adaptive advantages presented by *SEF1-VP16*-containing cells under iron-deprived (fig. 6*A*) and desiccation conditions (fig. 6*B*). Although the initial accumulation of nonfunctional binding events is likely neutral to the cell, these nonfunctional binding targets may provide immediate benefits for adaptation to changing environments when expressed by a mutant transcriptional factor or perturbed regulatory network. Our results provide direct evidence to support the model describing the evolutionary steps of prior plastic rewiring followed by a functional switch of a TF during network rewiring (Nocedal et al. 2017).

### Switches between Nonfunctional and Functional TF Binding: Proposed Models and Perspectives

The prevalence of nonfunctional TF-binding events may represent an ongoing stage in the pathway degeneration process before the TF completely loses its influence on these target genes or an initial stage of new regulatory network formation. Both scenarios can trigger plastic rewiring of the native gene regulatory network. The “degeneration” model hypothesizes that a TF was the primary regulator of gene A in the common ancestor (supplementary fig. S10*A*, Supplementary Material online). During evolution, TF transcriptional activity decreased possibly due to some environmental perturbations that potentiated changes in TF. To maintain the required expression level of gene A, evolutionary tinkering (i.e., compensatory evolution) enabled TF X to take over the regulation of gene A. This scenario is similar to cases of ribosomal gene regulation in yeasts (Lavoie et al. 2010). Ultimately, TF’s contribution to the expression of gene A becomes negligible, allowing mutations to accumulate in the TF-binding site so that the TF-binding ability is lost. In the “new regulation” model, gene A was originally controlled by transcription regulator X (supplementary fig. S10*B*, Supplementary Material online). During evolution, spontaneous mutations occurred in the promoter region of gene A that created a low-affinity TF-binding site, allowing the nonfunctional binding of TF. However, environmental changes could have increased demand for the gene A product, with the consequent selective force increasing the TF contribution by mutating the binding site to a high-affinity motif or by enhancing the transcriptional activity of TF. Through such changes, the reinforced TF might eventually outcompete original transcription regulator X, dominating regulation of gene A expression.

The proposed models are corroborated by the phenotypic assays of ortholog replacement lines. When we put Sef1 orthologs from other species into the *Lk-sef1*Δ strains, the complementation effects of these orthologs correlated with their heterologous transcriptional activation activities measured by the one-hybrid assay, that is, the higher the transcriptional activity is (supplementary fig. S11*A*, Supplementary Material online), the better the Sef1 orthologs can complement the defects of *Lk-sef1*Δ (supplementary fig. S11*B*, Supplementary Material online). Although these data cannot be used as direct evidence to indicate that Sef1 had changed its activity during evolution, it still reveals the possible physiological impact when a TF changes its transcriptional activity.

As observed in this study for the *SEF1-VP16* line, enhancing the activity of a TF enabled cells to gain novel phenotypes and, in this particular case, enabled better survival (fig. 6*A* and *B*). Moreover, 36 of the *SEF1-VP16*-induced genes are conserved genes unrelated to the TCA cycle and iron uptake, including *GPD1* (involved in glycerol synthesis and osmotic stress response), *HSP10* (involved in mitochondrial protein folding and sorting), *LEU5* and *MET17* (involved in amino acid biosynthesis), and *CRG1* (involved in lipid homeostasis). These changes may eventually lead to adaptive evolution if the cells encounter environmental fluctuations. Given that Sef1 has hundreds of binding sites in the genome, the repertoire of potential adaptive phenotypes is enormous, which may allow the cells to have additional rounds of plastic rewiring and functional switches in the Sef1 networks in the future.

Perturbations in the “hub” genes of a protein interaction network have been suggested to influence the overall phenotypic plasticity of organisms (Koubkova-Yu et al. 2018), allowing cells to gain novel adaptive potential unavailable to wild-type cells. The hub position of TFs in regulatory networks also suggests that their perturbations can have similar evolutionary impacts. However, TFs may exhibit greater flexibility than other hub components, so apart from changing activity or binding sites, a TF can change its regulatory patterns by interacting with other TFs (Mallick and Whiteway 2013) or expand its downstream targets by regulating another TF, as for the case in this study by which Lk-Sef1-VP16 regulates Lk-Aft1. Although several cases of transcriptional rewiring have been documented (Dalal and Johnson 2017; Sorrells et al. 2018; Britton et al. 2020), it will be interesting to establish how many such events have also led to altered evolutionary trajectories or novel adaptations.

## Materials and Methods

Isogenic strains were reconstructed and used for all experiments. All strains, plasmids, primers, genome resources, media, and chemicals are provided in supplementary tables S8–S12, Supplementary Material online. Complete details of the strains and plasmid constructions, phenotypic assays, beta-galactosidase assays, gene expression analyses, ChIP-seq analyses, bioinformatic analyses, statistical analyses, and supplementary figures and table legends are provided in Supplementary Material online.

## Supplementary Material

Supplementary data are available at *Molecular Biology and Evolution* online.

## Supplementary Material

msab192_Supplementary_Data
