# Supplementary material for: Plastic Rewiring of Sef1 Transcriptional Networks and the Potential of Nonfunctional Transcription Factor Binding in Facilitating Adaptive Evolution
Source: Mol Biol Evol. 2021 Jun 27;38(11):4732–47. doi: 10.1093/molbev/msab192 (PMC8557406; doi:10.1093/molbev/msab192)
Supplement: msab192_Supplementary_Data [file msab192_supplementary_data.zip › Hsu_MS_20210614+Fig_suppl_final.pdf]

Plastic rewiring of Sef1 transcriptional networks and the potential of non-functional transcription factor binding in facilitating adaptive evolution

Po-Chen Hsu*, Tzu-Chiao Lu, Po-Hsiang Hung, Yu-Ting Jhou, Ahmed A A Amine, Chia-Wei Liao, and Jun-Yi Leu *

* Correspondence:

Po-Chen Hsu (godshi2006@gmail.com)

Jun-Yi Leu (jleu@imb.sinica.edu.tw)

Supplementary Materials and Methods

Genome resources

Details of the genome sequences and annotations used in this study are listed in Table S11. Here, only the Bioproject accession numbers are shown, including *Lachancea kluyveri* (PRJNA1445), *Saccharomyces cerevisiae* (PRJNA260311, PRJNA324291), *Candida albicans* (PRJNA10701), *Hansenula polymorpha* (PRJNA60503), *Pichia pastoris* (PRJEA37871), *Kluyveromyces lactis* (PRJNA13835), and *Yarrowia lipolytica* (PRJNA295780). The *SEF1* orthologs were identified based on each genome annotation and then confirmed by the reciprocal BLAST strategy.

Strain and plasmid constructions

To avoid confusion, we have used the term “wild-type” to describe the “type strains” of all species used in our study, which may carry auxotrophic mutations or specific genetic manipulations, and they acted as reference strains for all comparative experiments. The strains used in this study are listed in Table S8. The plasmids and primers used for genetic manipulation are listed in Tables S9 and S10, respectively. All the DNA fragments used in cloning were PCR-amplified using Phusion High-Fidelity DNA polymerase (F530L, Thermo Fisher Scientific, USA). The PCR products and restriction digested products were purified by PCR cleanup or gel extraction using the AccuPrep® PCR/Gel Purification Kit (K-3037, Bioneer, Korea). All the plasmids were extracted and purified by the Presto™ Mini Plasmid Kit (PDH300, Geneaid, Taiwan). Successful strain constructions were confirmed by genomic DNA extraction as described previously (Hsu, et al. 2011), followed by PCR diagnostics using a homemade Taq DNA polymerase for genotyping.

For gene deletions, the DNA fragments of each deletion module consisting of a selection marker flanked by 5’ and 3’ homologous sequences to the target locus were created

by overlap-extension-PCR (Shevchuk, et al. 2004) (for *S. cerevisiae*, *L. kluyveri*, *K. lactis*, *H. polymorpha*, and *Y. lipolytica*), the SAT1 flipper method (Reuss, et al. 2004) (for *C. albicans* and *L. kluyveri*), or the Cre-ZeoR method (Pan, et al. 2011) (for *P. pastoris*). Briefly, each pair of 5’ and 3’ flanking regions for the target gene was PCR-amplified from the genomic DNA of each species and the fusion PCR was done by using Phusion High-Fidelity DNA polymerase (F530L, Thermo Fisher Scientific) with the help of ≥20–40 base-pair (bp) overlap sequences between the drug markers (KanMX6 (Wach, et al. 1997), HphMX4 (Goldstein and McCusker 1999), and NatMX4 (Goldstein and McCusker 1999)) and paired flanking sequences. For the SAT1 flipper system, the flanking sequences were fused with the SAT1-FLIP cassette in the pSFS2A plasmid by two rounds of ligation (T4 DNA ligase, M180A, Promega, USA) or by the In-Fusion® HD Cloning Kit (639650, ClonTech by Takara, Japan). For the Cre-ZeoR method, the *lox71*-Cre-ZeoR-*lox66* cassette was also fused with 5’ and 3’ flanking sequences by overlap-extension-PCR. To increase integration efficiency, about 1000 bp of flanking sequences were used for *Y. lipolytica SEF1* deletion. For integrations in other species, at least 300 bp sequences were used.

For gene reconstitution, replacement, and tagging in *L. kluyveri*, the SAT1 flipper method (Reuss, et al. 2004) was used. To create the *L. kluyveri SEF1* reconstitution plasmid, DNA fragments of the *SEF1* promoter (−1 to −638) and open reading frame (ORF) were PCR-amplified, restriction-digested, and ligated into the 5’ multiple cloning sites (MCS) of the SAT1-FLIP cassette between the KpnI-ApaI sites and the ApaI-XhoI sites, respectively. To create the *SEF1* ortholog replacement plasmids, the full-length *Ca-SEF1*, *Sc-SEF1*, *Hp-SEF1*, and *Pp-SEF1* ORFs were cloned into the reconstitution plasmid between the the ApaI-XhoI sites to replace the *Lk-SEF1* ORF. The native ApaI and XhoI sites on the *Hp-SEF1* ORF and the KpnI site on the *Pp-SEF1* ORF were synonymously mutated by overlap-extension-PCR-based site-specific mutagenesis (Sambrook and Russell 2001). To create the Sef1 C-terminal

tagging plasmids, the lexA, TAP, and VP16 (activation domain-only) sequences were amplified from pEG202 (ClonTech by Takara), pBS1479 (Puig, et al. 2001), and pC2HP (Stynen, et al. 2010), respectively, and cloned between the ApaI-XhoI sites with a 5' (Gly)3-Pro-(Gly)2 polylinker sequence in-frame and 3' STOP codons. The native SacI and SacII sites on the TAP sequences were synonymously mutated by overlap-extension-PCR-based site-specific mutagenesis (Sambrook and Russell 2001). Since an ApaI site is located very close to the VP16 N-terminal end, the VP16 fragment was ligated into pSFS2A and derived vectors by using the In-Fusion method. In the tagging plasmids, the 638-bp C-terminus of *SEF1* (+2492 to +3129 of the ORF without the STOP codon) was ligated into the pSFS2A vector between the KpnI-ApaI sites. In the *SEF1* ortholog tagging plasmids, the fragments made by fusing *Lk-SEF1* promoter with each *Ca-SEF1*, *Sc-SEF1*, *Hp-SEF1*, or *Pp-SEF1* ORF without the STOP codon was cloned into the tagging vector between the KpnI-ApaI sites. Each *SEF1* ortholog sequence was amplified from each replacement plasmid and the ApaI site locating between the *Lk-SEF1* promoter and each ORF was mutated to avoid undesired cutting during cloning. For both the reconstitution and tagging plasmids, the *L. kluyveri SEF1* 3'-UTR (terminator, +65 to +468 from STOP) sequence was ligated into the 3' MCS of the SAT1-FLIP cassette between the SacII-SacI sites. To transform cells, the KpnI-SacI-digested fragments from both the reconstitution and tagging plasmids were used in electroporation. Notably, because the *C. albicans MAL2* promoter on the SAT1-FLIP cassette is leaky in *L. kluyveri*, the *L. kluyveri* SAT1-FLIP transformants could only be selected and propagated in YPD+Nou10 plates during strain construction procedures. The integrated SAT1-FLIP cassette does not support growth of *L. kluyveri* cells in liquid broth with CloNat.

To create the lexA-operator-regulated LacZ reporter strains (SklexAOPlacZ strains) used in the one-hybrid assays, the *ScURA3-lexAOP-LacZ* fragments amplified from pSH18-34 (ClonTech by Takara) were fused with the upstream *L. kluyveri URA3* promoter (−1 to

−650 from ATG) and the downstream *L. kluyveri URA3* terminator (−15 to +585 from STOP codon) by overlap-extension-PCR. The product was integrated into the *URA3* locus of JYL1897 (*ura3*⁻) and transformants were selected by SC−Ura. To create the one-hybrid plasmids, the *ScADH1p-lexA-MCS-ScADH1t* fragments were amplified from pEG202 and ligated into pRS41H (Taxis and Knop 2006) between the KpnI-SacI sites to generate pRS41H-lexA-3. The *SEF1* and *SEF1-VP16* fragments with STOP codons were ligated in-frame into pRS41H-lexA-3 between the BamHI-NotI sites downstream of the lexA sequence to generate pRS41H-lexA-SkSef1-2 and pRS41H-lexA-SkSef1-VP16-1, respectively. The plasmid-based one-hybrid strains were created by transforming the SklexAOPlacZ-1 strain with these two plasmids and then selected in YPD+HGB. Notably, expression of N-terminal-tagged Sef1 was driven by the foreign *S. cerevisiae ADH1* promoter in the plasmid-based one-hybrid strains. In the integrated one-hybrid system, Sef1 was tagged with lexA at the C-terminus and expressed in the native locus.

To create the LacZ reporter plasmids used in the promoter assays, the DNA fragments of LacZ ORF-terminator were amplified from pSH18-34 (ClonTech by Takara) and ligated into pRS41H and pRS41K (Taxis and Knop 2006) between the ApaI-NotI sites by using the In-Fusion method to generate pRS41H-LacZ-1 and pRS41K-LacZ-1, respectively. To create the promoter-LacZ plasmids, each promoter sequence was PCR-amplified and cloned into the LacZ reporter plasmids between the KpnI-ApaI sites. The *IDH2* (SAKL0G03520g) and *KGD1* (SAKL0E08866g) promoters were fused into pRS41K-LacZ-1 by ligation. The *IDH1* (SAKL0E07876g) promoter was fused into pRS41H-LacZ-1 by ligation. The SAKL0B05940g and *FET3* (SAKL0B12056g) promoters were fused into pRS41H-LacZ-1 by the In-Fusion method. The promoter-LacZ strains of *IDH1*, *IDH2*, *KGD1*, and SAKL0B05940g were created by transforming JYL1897, SkSef1KA1, and SkSef1VP16NB10-1 with the corresponding plasmids. The *FET3*p-LacZ strains were created by transforming JYL1897,

SkSef1VP16NB10-1, SkAftKA22, and SkSef1VP16Aft1KA4. The transformants were selected in YPD+HGB or YPD+G418 medium.

To create the *ACO1* overexpression strains, DNA fragments of the *URA3* 5' flanking region (−1 to −-534), the *TEF2* promoter (-991 ~ +23) fused with the KpnI site-mutated *ACO1* ORF, and the *URA3* 3' flanking region (+110 to +585 from STOP codon) were PCR-amplified, restriction-digested, and ligated into the 5' or 3' MCS of the SAT1-FLIP cassette between the KpnI-ApaI sites, the ApaI-XhoI sites, or the SacII-SacI sites, respectively. To transform cells, the KpnI-SacI-digested fragments from the overexpression plasmids were used in electroporation. All subsequent procedures were the same as those mentioned above when using the pSFS2A-based strategy.

Electroporation

All yeast transformations were performed by using electroporation as described previously (Hsu, et al. 2011) with some modifications. Briefly, cells from 10 ml of overnight culture in YPD medium were collected by centrifugation (1500 *g*, 5 min, 25˚C; Eppendorf 5810R centrifuge, A-4-62 rotor, Eppendorf, Germany) and resuspended in sterile lithium buffer (8 ml of ddH_2O, 1 ml of 10x TE [100 mM Tris-Cl; 10 mM EDTA, pH 8], and 1 ml of 1 M lithium acetate [pH 7.5], 250 μl of 1 M DTT). After incubation at 25°C for 1 h with rotation at 50 rpm, the cells were washed sequentially with 30 ml of ice-cold ddH_2O and 5 ml of ice-cold 1 M sorbitol. The final electrocompetent cells were resuspended in 500 μl 1 M sorbitol and kept on ice until use. Approximately 100 ng plasmid or 1 μg linear DNA fragments were mixed with 100 μl electrocompetent cells, and the mixture was shocked with the Thermo Fisher Scientific BTX™ Gemini SC2 Electroporation System (2 mm-Electroporation Cuvettes, BL6222, Basic Life Bioscience Inc., Taiwan; exponential decay mode: 1800 V, 200 Ω, 25 μF). After electroporation, the cells were immediately washed with 1 ml of 1 M sorbitol, recovered in 1

ml YPD medium for 2.5 h at 25°C with end-over-end mixing at 60 rpm (Intelli-Mixer, RM-2L, Daigger Scientific, USA; F1 mode), and then plated on appropriate selective plates. The plates were incubated at 28˚C for 2–3 days. The selective plates for each selective marker are listed in Table S12.

Media, important chemicals, and growth conditions

All the media and important chemicals used in this study are listed with abbreviations in Table S12. All agar plates contained 2% agar unless indicated otherwise. The basal YPD medium and synthetic medium were prepared according to Fink et al. (Guthrie and Fink 1991). Some modifications in the recipe of the synthetic medium are noted in Table S12. All the cultures in the culture tubes were grown on a drum roller rotating at 50–80 rpm or in flasks with orbital shaking at 180 rpm. The normal growth temperature for each yeast species was 28–30˚C. Heat stress was applied at 39˚C, as indicated in the experiments.

Phenotypic assays

For spot assays, cells grown in YPD overnight at 28˚C were harvested by centrifugation (Eppendorf 5810R centrifuge; A-4-62 rotor; 1500 *g*; 5 min; 25°C) and serially diluted to the desired cell densities with sterile ddH_2O. Each dilution (10^7–10^3 CFU/ml) was spotted onto the agar plates (5 μl/spot, about 10^5–10^1 CFU/spot) and incubated at 28-30˚C or other temperatures as indicated. The plates were scanned every day using a scanner (EPSON PERFECTION V750 PRO) and images were recorded for 3−5 days as indicated.

For the growth curve assays, cells grown in YPD overnight at 28˚C were inoculated into 120 μl YPD medium in a 96-well plate (Tissue Culture Testplate 96 wells F-bottom, 92096, TPP, Switzerland) at a cell density of 0.2 OD_{600}/ml. Cell growth was measured at OD_{595} in a "2x2 multiple reads per well" mode every 12 min using a Tecan plate reader (Infinite 200 PRO,

Tecan, Switzerland). Tecan software Magellan Version 7.2 was used for data acquisition and analyses. For the Magellan method, the plate definition "[TPP96ft]-Tecan Plastic Products AG6 Flat Transparent" was selected. Each 12-min cycle included 3 min reading, 1 min shaking, 3 min standing, 1 min shaking, 3 min standing, and 1 min shaking. For the respiration-inhibited medium, 1 μg/ml Antimycin A was added into the YPD medium.

For the TTC reduction assays, cells grown in YPD overnight at 28˚C were harvested and diluted at cell densities of 1 OD_{600}/ml and 3 OD_{600}/ml with sterile ddH_2O. Then 5 μl of each cell suspension was spotted onto YPGly plates. The plates were incubated at 28˚C for 2 days. The plates were then overlaid with 20 ml autoclaved TTC agar (0.1% TTC, 2% agar in PBS [8 g/l NaCl, 0.2 g/l KCl, 1.44 g/l Na_2HPO_4, 0.24 g/l KH_2PO_4, pH 7.4]) and allowed to solidify for 30 min at room temperature. The plates were incubated at 28˚C for red color development. The plates were scanned after 2.5 and 8 h of color development using a scanner (EPSON PERFECTION V750 PRO).

For the competition assays, cells grown in YPD overnight at 28˚C were harvested and diluted at cell densities of 1 OD_{600}/ml with sterile ddH_2O. The cell density for each suspension was validated by 2-fold dilution and OD_{600} measurement. For competition assay in respiratory conditions, equal amounts of the reference strain (G418-resistant) and the test strain (G418-sensitive) were mixed at a cell density of 0.05 OD_{600}/ml (each strain at 0.025 OD_{600}/ml) in a 20-ml YPGly culture, and the two strains were grown at 30˚C continuously for 3 days. For competition in post-diauxic conditions, equal amounts of two strains were mixed at a cell density of 0.2 OD_{600}/ml (each strain at 0.1 OD_{600}/ml) in a 10-ml YPD culture and the two strains competed for 2 days for 5 cycles. Between each cycle, the existing competition culture was diluted 1000-fold into a new 10-ml YPD culture. For competition under low-iron conditions, equal amounts of two strains were mixed at a cell density of 0.2 OD_{600}/ml (each strain at 0.1 OD_{600}/ml) in a 10-ml YPD+200 μM BPS culture and then the two strains competed

continuously for 6 days. The frequency of G418-resistant cells was calculated by CFU counting on YPD plates and then on replicated YPD+G418 plates from 4–5 technical repeats.

For the desiccation assays, cells grown in YPD overnight at 28°C were harvested and diluted at cell densities of 10 OD_{600}/ml with sterile ddH_2O. Equal amounts of the reference wild-type strain (G418-resistant) and the test *SEF1-VP16* strain (G418-sensitive) were mixed at a cell density of 1 OD_{600}/50 μl (each strain at 0.5 OD_{600}/50 μl) in a 1.5-ml tube. The desiccated sample was prepared by spinning down cells, removing supernatants, sealing the opened tube with a sterile breathable sealing film (BF-400-S, AXYGEN, USA), and air-drying the cell pellets at 28 or 39°C for 24 h. The control samples were prepared by retaining the supernatants and keeping the tubes capped. After 24 h, the cells were rehydrated in 1 ml sterile ddH_2O for 1 h. The ratio of G418-resistant and -sensitive cells was calculated by CFU counting on YPD plates and then on replicated YPD+G418 plates from 4–5 technical repeats.

Beta-galactosidase assays

The LacZ expression levels for the one-hybrid assays and promoter assays were quantified using liquid β-galactosidase assays as described previously (Hsu, et al. 2011) with some modifications. Briefly, about 10 OD_{600} of mid-log-phase cells were resuspended in 1 ml Z-buffer with freshly added β-mercaptoethanol (β-ME) (0.06 M Na_2HPO_4, 0.04 M NaH_2PO_4, 0.01 M KCl, 1 mM $MgSO_4 \cdot 7H_2O$, 120 μl/30 ml β-ME, pH 7). Cells in 50–100 μl of suspension were lysed by adding 15 μl of 0.1% SDS and 30 μl chloroform and vortexing for 15 sec. Then, the suspensions of lysed cells were incubated with 200 μl of 4 mg/ml ONPG in potassium phosphate buffer (pH 7.0) at 37°C for 15 min. The reaction was stopped by adding 0.4 ml of 1 M Na_2CO_3. The cell debris was spun down (Eppendorf 5810R centrifuge; A-4-62 rotor; 12K *g*; 10 min; 25°C), and the absorbance of supernatants at 420 nm and 550 nm was determined with a spectrophotometer. LacZ levels are displayed as Miller units.

RNA extraction and DNase treatment

Total RNA was extracted by the phenol-chloroform method as described previously with some modifications (Hsu, et al. 2011). Briefly, mid-log-phase (1.0–3.0) cells from subcultures grown under different iron conditions or early-log-phase (0.5–1.0 OD_{600}/ml) cells from subcultures grown under YPD versus YPGly conditions were harvested by centrifugation (Eppendorf 5810R centrifuge; A-4-62 rotor; maximum speed; 5 min; 25°C), washed once with ddH_2O, and stored at −80°C. Cells (~10 OD_{600}) were thawed on ice and resuspended in 500 μl of nuclease-free and ice-cold lysis buffer (0.1 M Tris-Cl [pH 7.5], 0.1 M LiCl, 2% β-ME, 0.01 M EDTA, and 5% SDS). The mixtures were transferred to a microcentrifuge tube containing ice-cold 500 μl PCIA (Phenol:chloroform:isoamyl alcohol = 25:24:1, pH 4.5; 0.1% 8-Hydroxyquinoline) and 0.3 g glass beads (11079105, BioSpec Products, USA). The cells were lysed by vortexing them at maximum speed for 5 min. Supernatants were obtained by centrifugation (Eppendorf 5424R centrifuge; FA-45-24-11 rotor; maximum speed; 10 min; 4°C). Each aqueous phase was extracted from two more rounds of centrifugation following the addition of 200 μl ice-cold PCIA and mixing for 3 min. Each of the final aqueous phases (350–400 μl) was transferred into 1 ml of 99% ethanol for precipitation with mild mixing for 2 h. Nucleic acid pellets were spun down (maximum speed), washed with 70% ethanol, air-dried for 5 min, and then resuspended in 100 μl nuclease-free water (UltraPure™ DNase/RNase-Free Distilled Water, 10977015, Invitrogen by Thermo Fisher Scientific).

DNA contaminants in the total RNA were removed with the TURBO DNA-free™ kit (AM1907, Ambion, Invitrogen by Thermo Fisher Scientific) by treating 40 μg of total RNA at 37˚C for 40 min with 2 μl of TURBO DNase in a 50-μl reaction. The DNase was removed by treating the reaction mixture with 5 μl of inactivation reagent beads for 15 min at room temperature with mild vortexing every 5 min. The purified RNA in the supernatants was

collected by spinning down the beads and quantified by measuring the OD_{260} using an ND-1000 spectrophotometer (Nanodrop Technology, USA). The final RNA quality was checked using a Bioanalyzer 2100 instrument (Agilent Technologies, USA) with an RNA 6000 Nano LabChip kit (Agilent Technologies).

Reverse transcription (RT) and quantitative PCR (qPCR) analyses

For cDNA synthesis, 2.0 μg pure RNA per 20-μl reaction was reverse-transcribed by using the High-Capacity cDNA Reverse Transcription Kit (4368813, Applied Biosystems by Thermo Fisher Scientific) according to the manufacturer's instructions with some modifications. We replaced the random primers provided by the kit with synthesized nuclease-free oligo$(dT)_{18}$ primers at a concentration of 0.5 μg per 20-μl reaction. Moreover, the RNase inhibitors (Y9240L, Enzymatics by QIAGEN, Germany) were added at a concentration of 1 μl per 20-μl reaction. The reverse transcription reactions were performed with one cycle at 25°C for 10 min, followed by 2 h incubation at 42°C, and then 5 min heat-inactivation at 85°C.

Real-time qPCR was performed with the model 7500 Fast Real-Time PCR System and the model QuantStudio™ 12K Flex Real-Time PCR System (Applied Biosystems by Thermo Fisher Scientific). Primers used for qPCR are listed in Table S10. For some of the members in a gene family, the specific qPCR primers were designed to recognize the 3'-UTR. Briefly, each 20-μl reaction mixture contained 100 ng cDNA, 150 nM (each) primers, and 10 μl Fast SYBR™ Green Master Mix (4385612, Applied Biosystems by Thermo Fisher Scientific). The reactions were performed with one cycle at 95°C for 10 min followed by 40 repeated cycles at 95°C for 15 s and 60°C for 1 min. The *L. kluyveri ACT1* transcripts (SAKL0B00968g) were used as an endogenous control for qPCR. The average $\Delta\Delta C_T$ and standard deviation were determined from at least three technical repeats. The relative fold-change of each gene is shown according to the $2^{-\Delta\Delta CT}$ method.

Gene expression microarray analyses

For RNA preparations, cells were grown in the YPD medium at 28°C overnight and subsequently diluted into each indicated medium at cell densities of 0.2 OD_{600}/ml for YPD versus YPGly conditions or 0.5 OD_{600}/ml for iron conditions. After 5.5 h of incubation at 30°C, cells were harvested and RNA was purified as described above. For each strain and condition, total RNA was extracted from at least 3 to 4 biological replicates.

For Alexa dye-labeled cDNA synthesis, the SuperScript™ Plus Indirect cDNA Labeling System (L101402, Invitrogen by Thermo Fisher Scientific) was used according to the manufacturer's instructions. Briefly, 10 μg of DNase-treated RNA was reverse-transcribed using the anchored oligo[$(dT)_{20}$-VN] primers and the cDNA was labeled with aminoallyl-dUTP followed by column purification (28106, QIAquick DNA Purification Kit, QIAGEN). The aminoallyl-cDNA was then labeled with Alexa Flour® 555 dye (A-32756, Invitrogen by Thermo Fisher Scientific) and purified using a QIAGEN column (28106, QIAquick DNA Purification Kit, QIAGEN). The Agilent Gene Expression Hybridization Kit (5188-5242, Agilent Technologies) was used for hybridization according to the manufacturer's instructions. Briefly, 16 μl of Alexa dye-labeled cDNA in water was denatured at 98°C for 3 min in a reaction volume of 40 μl containing 4 μl Agilent 10x blocking Agent and 20 μl of Agilent 2x GEx Hybridization Buffer HI-RPM. Denatured dye-labeled cDNAs were subsequently hybridized to a custom *L. kluyveri* 8x15K microarray (Agilent-063206) at 65°C and rotated at 10 rpm on an Agilent hybridizer for 17 h. After hybridization, the microarrays were washed using the Agilent Gene Expression Wash Kit (5188-5327, Agilent Technologies). Briefly, microarrays were washed for 1 min at room temperature with GE Wash Buffer 1 and 1 min at 37°C with GE Wash buffer 2, and then dried immediately by brief centrifugation. Slides were scanned at 535 nm (for Alexa Flour® 555 dye) on an Agilent DNA Microarray Scanner (US9230696)

using the one color scan setting for 8x15k array slides (scan area: 61 x 21.6 mm; scan resolution: 5 μm; dye channel: green; green PMT: 100%; tiff: 16 bit; XDR: 0.10). Scanned images were analyzed with Feature Extraction 10.5.1.1 Software (Agilent Technologies) using default parameters (protocol: GE1_105_Dec08 and grid: 023206_D_F_20131222; provided by the IMB Genomics Core Lab, Academia Sinica, Taiwan) to obtain the background-subtracted and spatially detrended processed signal intensities. Features flagged in the Feature Extraction Software as "feature non-uniform outliers" were excluded.

To design gene probes, sequences of 5321 *L. kluyveri* transcripts of strain CBS3028 were extracted from the outdated Genolevures genome database (Sherman, et al. 2004; Martin, et al. 2011) (previously http://www.genolevures.org; no longer maintained) and uploaded to Agilent eArray. The genome of CBS3028 with updated annotations is now maintained at GRYC (Genome Resources for Yeast Chromosomes; http://gryc.inra.fr), which is part of iGenolevures web resources (http://bim.i2bc.paris-saclay.fr/igenolevures/index.php). The probes were analyzed by a base composition methodology. Duplicate sequences were removed, resulting in the generation of 10596 probes (8 genes with 1 BC1 probe; 5164 genes with 2 BC1 probes; 45 genes with 1 BC1 probe and 1 BC2 probe; 34 genes with 1 X-hyb probe). The probes were printed by in situ synthesis on each array, with an 8-by-15,000 format (Agilent Technologies). All designs were done by Welgene Biotech Co. Ltd. (https://www.welgene.com.tw).

For microarray data analyses, we used GeneSpring GX 11.5 software (Agilent Technologies). Raw expression levels were normalized to 75th percentile intensity. The higher signal intensities from one of the two probes were taken as the expression data for each gene according to the best probe list (Table S13). Probability scores were calculated with a default unpaired t-test of the ratio of median values from three or four biological replicates. Gene expression differences with p values ≤ 0.05 and 1.5 fold-change were

considered significantly different. The Benjamini-Hochberg corrections for p-values were performed for iron-related gene expression analyses. Specifically, since we describe pervasive non-functional binding events of Lk-Sef1 in our Results, the multiple testing correction was not performed to lessen statistical stringency so as to include as many differentially expressed genes as possible in the comparison between Sef1-sensitive genes and Sef1-bound genes.

Chromatin immunoprecipitation (ChIP)

Cells of SkSef1TAPNB1-1 (*SEF1-TAP*) and JYL1897 (no tag) were grown overnight in YPD medium at 30°C and subsequently diluted into each indicated medium at a cell density of 0.2 OD_{600}/ml. After 5.5 h of incubation at 30°C, cultures of mid-log-phase cells (1 OD_{600}/ml in YPD and 0.5 OD_{600}/ml in YPGly) were fixed with 1% formaldehyde at 25˚C for 20 min with shaking at 180 rpm, and then quenched with 125 mM glycine at 25˚C for 10 min with shaking at 180 rpm. All subsequent steps were performed in an ice-cold or 4˚C environment. Cells were harvested, washed twice with TBS (20 mM Tris-Cl, pH 7.5; 150 mM NaCl), and stored at −80˚C until use.

To break cells, 500 OD_{600} of cells were thawed and resuspended in 4 ml of FA buffer (50 mM HEPES, pH 7.5; 140 mM NaCl; 0.1% SDS; 1 mM EDTA; 1% Triton X-100; 0.1% Deoxycholate, Na salt) with 1/100 PIC (Protease inhibitor cocktail Set IV, in DMSO; 539136, Merck, Germany) and 1 mM phenylmethanesulfonyl fluoride (PMSF, P7626, Sigma, USA). The cell suspension was divided into four 2-ml breaking tubes (Biospec, 10832, Microtube 2 ml with cap) containing 1 ml volume of glass beads (11079105, BioSpec Products). Lysis was performed by three cycles of 5-min beating, followed by 1-min chilling on ice (Biospec Mini-BeadBeater-16). Cell lysates and glass beads were separated by punching a hole in the bottom of the tube with a red-hot 18G needle and subjecting it to slow centrifugation (500 *g*,

3 min, 4˚C; Eppendorf 5810R centrifuge, A-4-62 rotor). The collected lysates were combined into a 15-ml centrifuge tube and washed with two cycles of 5 ml FA buffer followed by centrifugation (12K rpm, 5 min, 4˚C; Eppendorf 5810R centrifuge, F-34-6-38 rotor). The washed lysates were completely resuspended in 2 ml FA buffer with 1/100 PIC and 1 mM PMSF in a new 15-ml centrifuge tube (CFT011150, BIOFIL). Chromatin was sheared by sonication in a Bioruptor water-bath sonicator (Diagenode, USA) with a 15 ml tube-chip unit (High intensity; 30 sec on, 30 sec off, 15 min/cycle; total 3 cycles) at 4°C. The sheared lysate was centrifuged at 12000 *g* for 10 min at 4°C (Eppendorf 5810R centrifuge, F-34-6-38 rotor). About 25 μl of supernatant corresponding to 5 OD_{600} of cells was collected, mixed with 200 μl TES buffer (50 mM Tris-Cl, pH 8; 10 mM EDTA, pH 8; 1% SDS) plus 175 μl TE buffer (10 mM Tris-Cl; 1 mM EDTA, pH 8), and stored at -20˚C as the input DNA.

Immunoprecipitation was performed by incubating all remaining sheared chromatins with 300 μl Dynabeads® Pan Mouse IgG (11041, human anti-mouse IgG, Invitrogen by Thermo Fisher Scientific) in 6 ml of FA buffer with 1/100 PIC and 1 mM PMSF. The binding was performed in a 15-ml tube with mixing using an end-over-end rotator (Intelli-Mixer, RM-2L, F1 mode, 30 rpm) at 4˚C for 16 h. The magnetic beads were anchored with DynaMag™-2 Magnet and the bound complexes were washed four times with 1 ml of each wash buffer for 5 min [FA buffer × 1, high-salt FA buffer (50 mM HEPES, pH 7.5; 500 mM NaCl; 0.1% SDS; 1 mM EDTA; 1% Triton X-100; 0.1% Deoxycholate, Na salt) × 1, DOC buffer (10 mM Tris·Cl; 1 mM EDTA, pH 8; 250 mM LiCl; 0.5% IGEPAL® CA-630; 0.5% Deoxycholate, Na salt) × 1, and TE buffer × 1] with mixing (Intelli-Mixer, RM-2L, F1 mode, 30 rpm) at room temperature. The bound complexes were eluted twice by heating at 65˚C in 200 μl TES buffer for 20 min and then in 200 μl TE buffer for 10 min. The two eluates were pooled as the ChIP-DNA. To remove RNA, both the input DNA and ChIP-DNA (~400 μl each) were treated with 5 μl of 10 mg/ml RNase A (R5503, Sigma; 100 mM Tris-Cl, pH 7.4) at 37˚C for 30 min. For

de-crosslinking, both the RNase-treated input DNA and ChIP-DNA were treated with 40 μl of 20 mg/ml Proteinase K (1.24568.0500, Merck; in 50 mM Tris-Cl, pH 8) at 42°C for 1 h and then at 65°C for 16 h. De-crosslinked DNA was purified using the QIAquick DNA Purification Kit (28106, QIAGEN) according to the manufacturer's instructions, with a modification of a two-cycle wash step using the PE buffer. Final ChIP-DNA and input DNA were eluted with 50 μl EB buffer. DNA concentrations were measured by using the Qubit™ dsDNA HS Assay Kit (Q32854, Invitrogen by Thermo Fisher Scientific).

ChIP-seq analyses

ChIP-seq analysis was performed on three biological replicates of YPD- and YPGly-grown cells. The average fragment length of sonicated fragments was 150–1000 bp. For each condition, libraries were prepared from 3.96–20 ng of ChIP-DNA or input DNA using the KAPA LTP Library Preparation Kit for Illumina® platforms (KK8232, KAPA Biosystems by Roche, Switzerland) according to the manufacturer's instructions. Notably, after adapter ligation, a double-sided size selection of 250–450 bp for adapter-ligated fragments was performed using the KAPA Pure Beads system (KK8000/07983271001, KAPA Biosystems by Roche). Size-selected fragments were then PCR-amplified for 12 cycles. The size of each library was assessed using a Bioanalyzer 2100 instrument (Agilent Technologies) with the High Sensitivity DNA Kit (Agilent Technologies). The concentration of each library was quantified by using the Qubit™ dsDNA HS Assay Kit (Q32854, Invitrogen by Thermo Fisher Scientific) and qPCR. Single-read sequencing (75 bp) of the libraries was performed using the NextSeq 500/550 high output reagent kit V2_75 cycle (FC-404-2005, Illumina, USA) on an Illumina NextSeq500 sequencer. The sequencing was performed at a depth of ~40 million reads (~200-fold coverage to the genome).

Quality control and adaptor trimming of sequencing reads were processed in FastQC

version 0.11.8 (https://www.bioinformatics.babraham.ac.uk/projects/fastqc/) and Trimmomatic version 0.36 (Bolger, et al. 2014) with the parameters “2:30:10 LEADING:3 TRAILING:3 SLIDINGWINDOW:4:15 HEADCROP:3 CROP:68”. Trimmed reads were mapped to the *L. kluyveri* CBS 3082 genome using Bowtie2 version 2.3.3 (Langmead and Salzberg 2012). To determine which genomic regions are enriched for Sef1 binding, Bowtie2-mapped BAM files were assessed for ChIP peaks using SPP version 1.13 (Kharchenko, et al. 2008) with a false discovery rate (FDR) of 0.05. The Sef1 binding peaks were compared and merged among three biological replicates by Diffbind version 2.10.0 (Ross-Innes, et al. 2012). Only peaks detected in all three biological repeats (895 peaks out of 1074 total peaks) were used for the downstream analysis. As the negative control, no peak was called from the ChIP DNA of non-tagged Sef1 strain (JYL1897) using the same workflow.

For the peak-to-gene assignments, the peaks were assigned to specific genes on the basis of the location of each peak center. Only peaks with centers located within the region −2000 to +200 from the ATG of the nearest target genes were assigned. A total of 506 (57%) peak centers were located at a distance of <500 bp from ATG, 146 peak centers were located at a distance of between 500 and 1000 bp from ATG, 154 peak centers were located at a distance of between 1000 and 2000 bp from ATG, and 89 peak centers were located at a distance of >2000 bp from ATG (Table S3). Overall, of 895 peaks, 806 were assigned to target genes. To increase the accuracy of target gene identification, three biological replicates were performed for each condition. Only genes with Sef1-binding peaks in all three replicates were considered as bona fide targets, even for the weak bound targets (see Fig. S12 as examples)

MEME and MEME-ChIP analyses for motif discovery

Peak regions were defined as ±125 bp from each peak center (total 250 bp). The 250-bp peak sequences of the top 450 peaks bound by *L. kluyveri* Sef1 and enriched under the

YPGly condition were submitted to MEME-ChIP Version 4.12.0 (http://meme-suite.org/tools/meme-chip) (Machanick and Bailey 2011) for motif analysis using the following settings: Minimum width = 6, Maximum width = 30, Count of motifs = 10, Motif site distribution = zero or one occurrence per sequence. All other parameters were the default settings. The raw result produced by MEME-suite is available in supplementary Data File S1 as a compressed folder upon request. To repeat *C. albicans* Sef1 binding motif discovery, the MEME analysis was performed according to a previous study (Chen, et al. 2011).

FIMO analyses for motif scan

Motif 3 identified from MEME, as described above, was adopted as the *L. kluyveri* Sef1 binding motif and submitted to FIMO Version 5.0.2 (Grant, et al. 2011) built in the MEME-suite (http://meme-suite.org/tools/fimo) to scan the 895 peak sequences bound by Lk-Sef1. As a preliminary analysis, a p-value < 0.1 was applied to obtain a full list of scanned motifs. The p-value represents the probability of a random sequence of the same length as the motif matching that position of the sequence with a score at least as good as the motif. Subsequent manual filtering by changing the p-value cut-off from 0.1 to 0.0001 identified high-confidence motifs. The raw results are available in supplementary Data File S2 as a compressed folder upon request, and the number of motifs identified based on different p-value cut-offs is shown in Fig. S4.

Gene ontology (GO) analyses

For *L. kluyveri* GO analyses, gene ontology information borrowed from *S. cerevisiae* (SGD, http://www.yeastgenome.org; GO Term Finder, version 0.86) was used to analyze the *L. kluyveri* genes. Briefly, the *L. kluyveri* genes were converted to *S. cerevisiae* orthologs using a cross-reference table (Table S14). The cross-reference table was created by combining the

L. kluyveri annotation in GRYC (http://gryc.inra.fr), the Lk-to-Sc orthology from a previous study (Brion, et al. 2015), and the syntenic contents between *L. kluyveri* and *S. cerevisiae* in YGOB (Byrne and Wolfe 2005). The *L. kluyveri* genes in the input list without a clear *S. cerevisiae* ortholog (~10%) were omitted from the GO analyses. The enriched GO terms with corrected p-values < 0.0001 were considered significant. We did not correct for the genetic background because *L. kluyveri* and *S. cerevisiae* share similar gene numbers in their genomes (5000–6000 genes) and the cut-off for corrected p-values is so stringent that background correction would not have changed the results and altered our conclusions.

Phylogenetic analysis

Sef1 orthologous sequences from *S. cerevisiae* (YBL066C), *L. kluyveri* (SAKL0F12342g), *K. lactis* (KLLA0E20307g), *H. polymorpha* (HPODL_03022), *P. pastoris* (PP7435_Chr3-0266), *C. albicans* (orf19.3753), and *Y. lipolytica* (YALI1_D06408g) were aligned in MUSCLE, and a phylogenetic tree was built using the ML algorithm with bootstrap support values from 1000 replicates. The tree with the highest log likelihood (-12960.07) is shown (Fig. S2D). This analysis involved seven amino acid sequences. There were a total of 1231 positions in the final dataset. Alignments and evolutionary analyses were conducted in MEGA X (Kumar, et al. 2018).

Statistical analysis

Details of statistical analyses are presented in the main text or corresponding figure legends. Error bars represent standard deviations. Statistical significance tests were carried out using the Student's t-test in Excel 2016 or the default functions packaged in each analysis tool.

Data availability

The microarray data have been submitted to the Gene Expression Omnibus website (http://www.ncbi.nlm.nih.gov/geo/) under accession numbers GSE130296, GSE130297, and GSE130300. The processed results are shown in Tables S1, S2, and S4-S7. The ChIP-seq data have been submitted to the Gene Expression Omnibus website (http://www.ncbi.nlm.nih.gov/geo/) under accession number GSE148243. The processed results are shown in Table S3.

Supplementary References

Bolger AM, Lohse M, Usadel B. 2014. Trimmomatic: a flexible trimmer for Illumina sequence data. Bioinformatics 30:2114-2120.

Brion C, Pflieger D, Friedrich A, Schacherer J. 2015. Evolution of intraspecific transcriptomic landscapes in yeasts. Nucleic Acids Res 43:4558-4568.

Byrne KP, Wolfe KH. 2005. The Yeast Gene Order Browser: combining curated homology and syntenic context reveals gene fate in polyploid species. Genome Res 15:1456-1461.

Chen C, Pande K, French SD, Tuch BB, Noble SM. 2011. An iron homeostasis regulatory circuit with reciprocal roles in Candida albicans commensalism and pathogenesis. Cell Host Microbe 10:118-135.

Goldstein AL, McCusker JH. 1999. Three new dominant drug resistance cassettes for gene disruption in Saccharomyces cerevisiae. Yeast 15:1541-1553.

Grant CE, Bailey TL, Noble WS. 2011. FIMO: scanning for occurrences of a given motif. Bioinformatics 27:1017-1018.

Guthrie C, Fink GR. 1991. Guide to yeast genetics and molecular biology. San Diego: Academic Press.

Hsu PC, Yang CY, Lan CY. 2011. Candida albicans Hap43 is a repressor induced under low-iron conditions and is essential for iron-responsive transcriptional regulation and virulence. Eukaryot Cell 10:207-225.

Kharchenko PV, Tolstorukov MY, Park PJ. 2008. Design and analysis of ChIP-seq experiments for DNA-binding proteins. Nat Biotechnol 26:1351-1359.

Kumar S, Stecher G, Li M, Knyaz C, Tamura K. 2018. MEGA X: Molecular Evolutionary Genetics Analysis across Computing Platforms. Mol Biol Evol 35:1547-1549.

Langmead B, Salzberg SL. 2012. Fast gapped-read alignment with Bowtie 2. Nat Methods 9:357-359.

Machanick P, Bailey TL. 2011. MEME-ChIP: motif analysis of large DNA datasets. Bioinformatics 27:1696-1697.

Martin T, Sherman DJ, Durrens P. 2011. The Genolevures database. C R Biol 334:585-589.

Pan R, Zhang J, Shen WL, Tao ZQ, Li SP, Yan X. 2011. Sequential deletion of Pichia pastoris genes by a self-excisable cassette. FEMS Yeast Res 11:292-298.

Puig O, Caspary F, Rigaut G, Rutz B, Bouveret E, Bragado-Nilsson E, Wilm M, Seraphin B. 2001. The tandem affinity purification (TAP) method: a general procedure of protein complex purification. Methods 24:218-229.

Reuss O, Vik A, Kolter R, Morschhauser J. 2004. The SAT1 flipper, an optimized tool for gene disruption in Candida albicans. Gene 341:119-127.

Robinson JT, Thorvaldsdóttir H, Winckler W, Guttman M, Lander ES, Getz G, Mesirov JP. 2011. Integrative genomics viewer. Nature Biotechnology 29:24-26.

Ross-Innes CS, Stark R, Teschendorff AE, Holmes KA, Ali HR, Dunning MJ, Brown GD, Gojis O, Ellis IO, Green AR, et al. 2012. Differential oestrogen receptor binding is associated with clinical outcome in breast cancer. Nature 481:389-393.

Sambrook J, Russell DW. 2001. Molecular cloning : a laboratory manual. Cold Spring Harbor, N.Y.: Cold Spring Harbor Laboratory Press.

Sherman D, Durrens P, Beyne E, Nikolski M, Souciet JL, Genolevures C. 2004. Genolevures: comparative genomics and molecular evolution of hemiascomycetous yeasts. Nucleic Acids Res 32:D315-318.

Shevchuk NA, Bryksin AV, Nusinovich YA, Cabello FC, Sutherland M, Ladisch S. 2004. Construction of long DNA molecules using long PCR-based fusion of several fragments simultaneously. Nucleic Acids Res 32:e19.

Stynen B, Van Dijck P, Tournu H. 2010. A CUG codon adapted two-hybrid system for the pathogenic fungus Candida albicans. Nucleic Acids Res 38:e184.

Taxis C, Knop M. 2006. System of centromeric, episomal, and integrative vectors based on drug resistance markers for Saccharomyces cerevisiae. Biotechniques 40:73-78.

Vakirlis N, Sarilar V, Drillon G, Fleiss A, Agier N, Meyniel J-P, Blanpain L, Carbone A, Devillers H, Dubois K, et al. 2016. Reconstruction of ancestral chromosome architecture and gene repertoire reveals principles of genome evolution in a model yeast genus. Genome research 26:918-932.

Wach A, Brachat A, Alberti-Segui C, Rebischung C, Philippsen P. 1997. Heterologous HIS3 marker and GFP reporter modules for PCR-targeting in Saccharomyces cerevisiae. Yeast 13:1065-1075.

Supplementary Figures and Tables

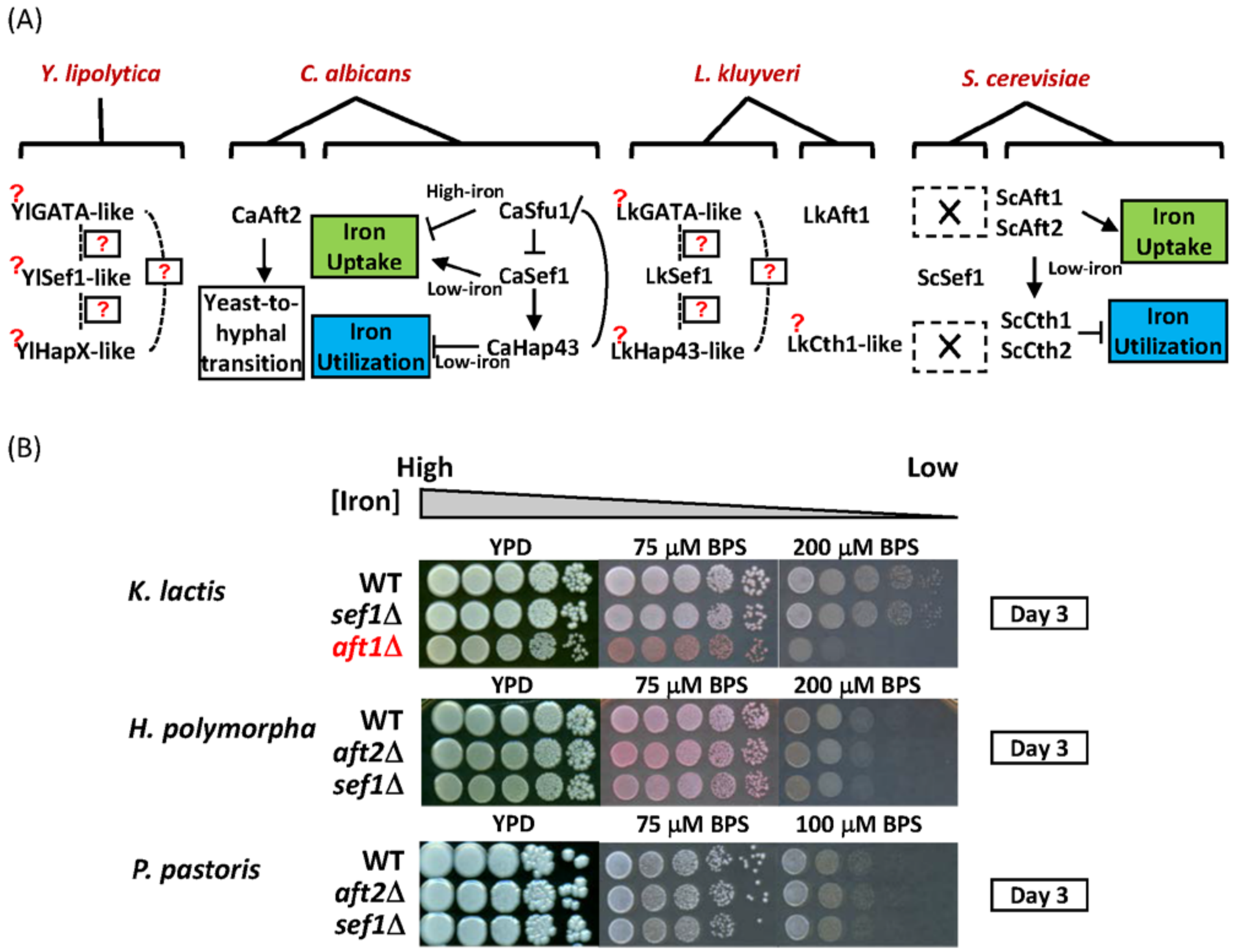

Figure S1. Growth of *sef1*Δ mutants under iron-deprived conditions. (A) Simplified regulatory networks of Sef1 in *Y. lipolytica*, *C. albicans*, *L. kluyveri*, and *S. cerevisiae*. Biological functions highlighted in green and blue are the same as those used in Fig. 1A. (B) Iron-dependent growth of *sef1*Δ in another protoploid yeast, *K. lactis*, and the methylotrophic yeasts *H. polymorpha* and *P. pastoris*. The mutant showing strong growth defects under iron-deprived conditions has been placed at the bottom of each panel and is highlighted in red. The indicated concentration of BPS was added into YPD plates to create iron-deprived conditions. All plates were incubated at 28˚C.

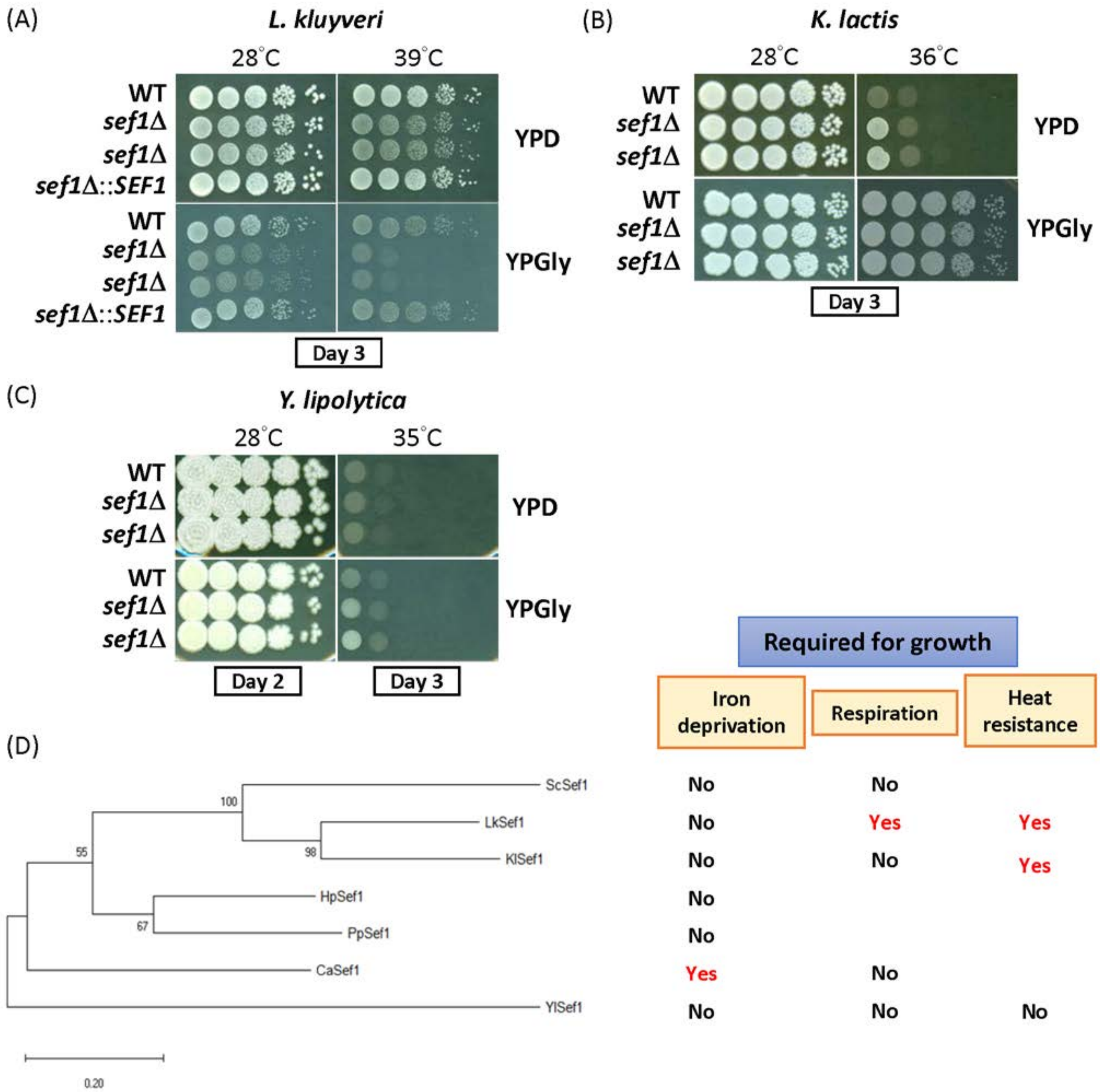

Figure S2. Growth of *sef1*Δ mutants under heat-stressed respiratory conditions. The growth of *sef1*Δ mutants in (A) *L. kluyveri*, (B) *K. lactis*, and (C) *Y. lipolytica* were assayed under more stringent (39°C) respiratory conditions. Two independent clones of the *sef1*Δ mutant were assessed. The *Lk-sef1*Δ cells show mild or severe growth defects under normal (28˚C) or more stringent (39˚C) respiratory conditions, respectively, with 39°C almost being the maximum permissive temperature that wild-type *L. kluyveri* can grow on agar plates. The *Kl-sef1*Δ cells only show mild heat-resistance under the fermentative condition (YPD+36˚C).

The *Yl-sef1*Δ cells show no fitness defect. The 35 and 36°C are the restrictive temperatures to the wild-type strains of *K. lactis* and *Y. lipolytica*, respectively. (D) Phylogeny of Sef1 based on amino acid sequences. Sef1 orthologous sequences from *S. cerevisiae* (Sc), *L. kluyveri* (Lk), *K. lactis* (Kl), *H. polymorpha* (Hp), *P. pastoris* (Pp), *C. albicans* (Ca), and *Y. lipolytica* (Yl) were analyzed. All sequences were aligned in MUSCLE and a phylogenetic tree was built using the ML algorithm with bootstrap support values from 1000 replicates. All analyses were done in MEGA-X. The essentiality of Sef1 orthologs in each yeast species for cell growth in the conditions tested in this study is noted at the right side of the phylogenetic tree.

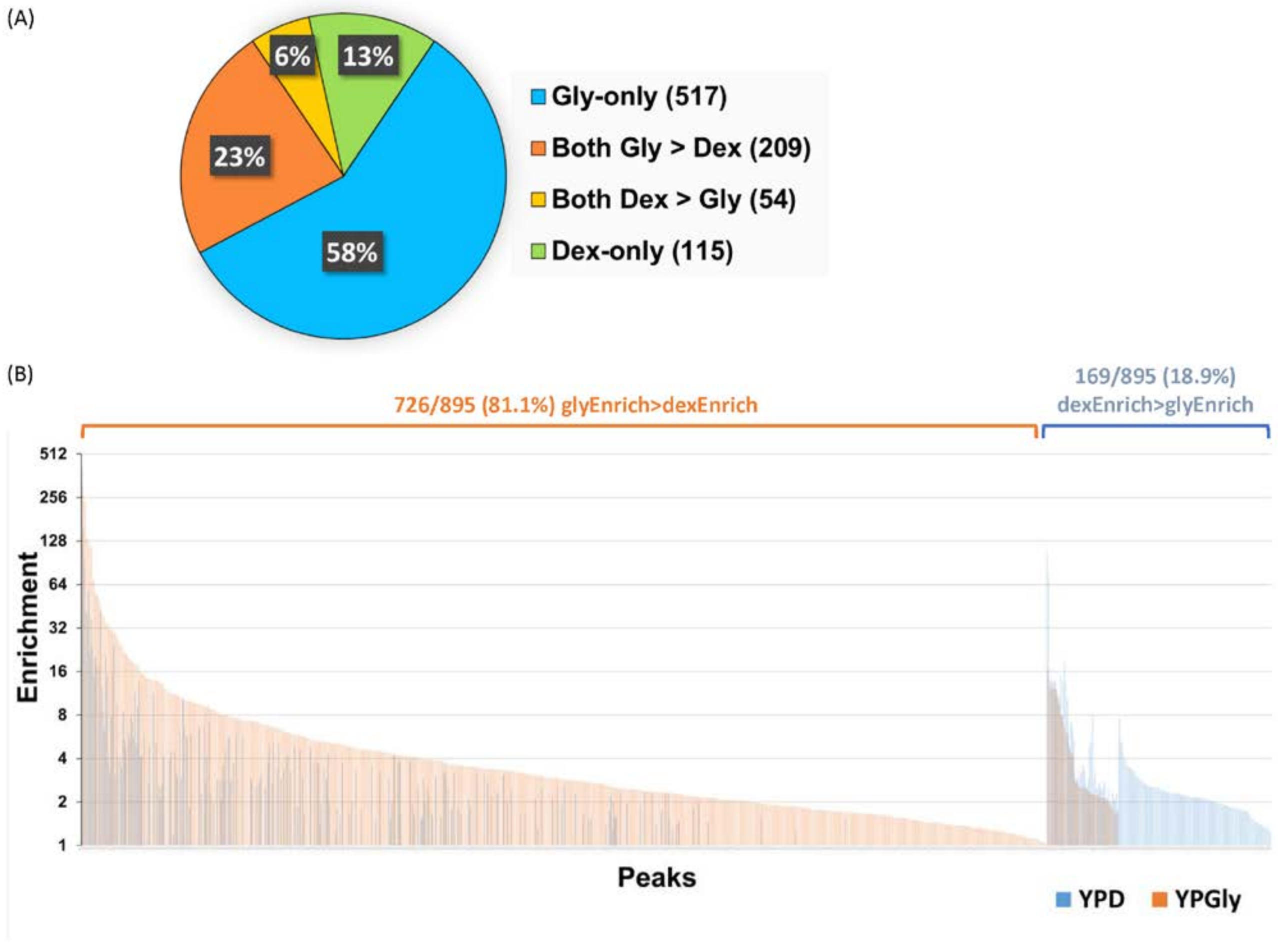

Figure S3. ChIP-seq analyses for *L. kluyveri* Sef1 under both YPD and YPGly conditions. (A) The number and frequency of each group of Sef1-bound peaks called from ChIP-seq. Each group is classified according to the enrichment values (glyEnrich and dexEnrich). The peaks from each set were identified from convergent peaks among three biological repeats. A total of 895 (517 + 209 + 54 +115) peaks were identified. (B) Peak ranking according to enrichment values. All 895 peaks (Peak_N, x-axis) are ranked based on ChIP enrichment values (y-axis) in descending order. For each peak, two enrichment values have been paired and depicted (dexEnrich：YPD (in blue); glyEnrich：YPGly (in orange)). If a peak was not called in all three biological replicates or was absent from one of the biological replicates in either of two conditions, the enrichment value of the peak under that condition was assigned as “zero”. Furthermore, peak sets have been sorted according to two criteria： glyEnrich > dexEnrich (726 peaks, left along the x-axis) and dexEnrich > glyEnrich (169 peaks,

right along the x-axis).

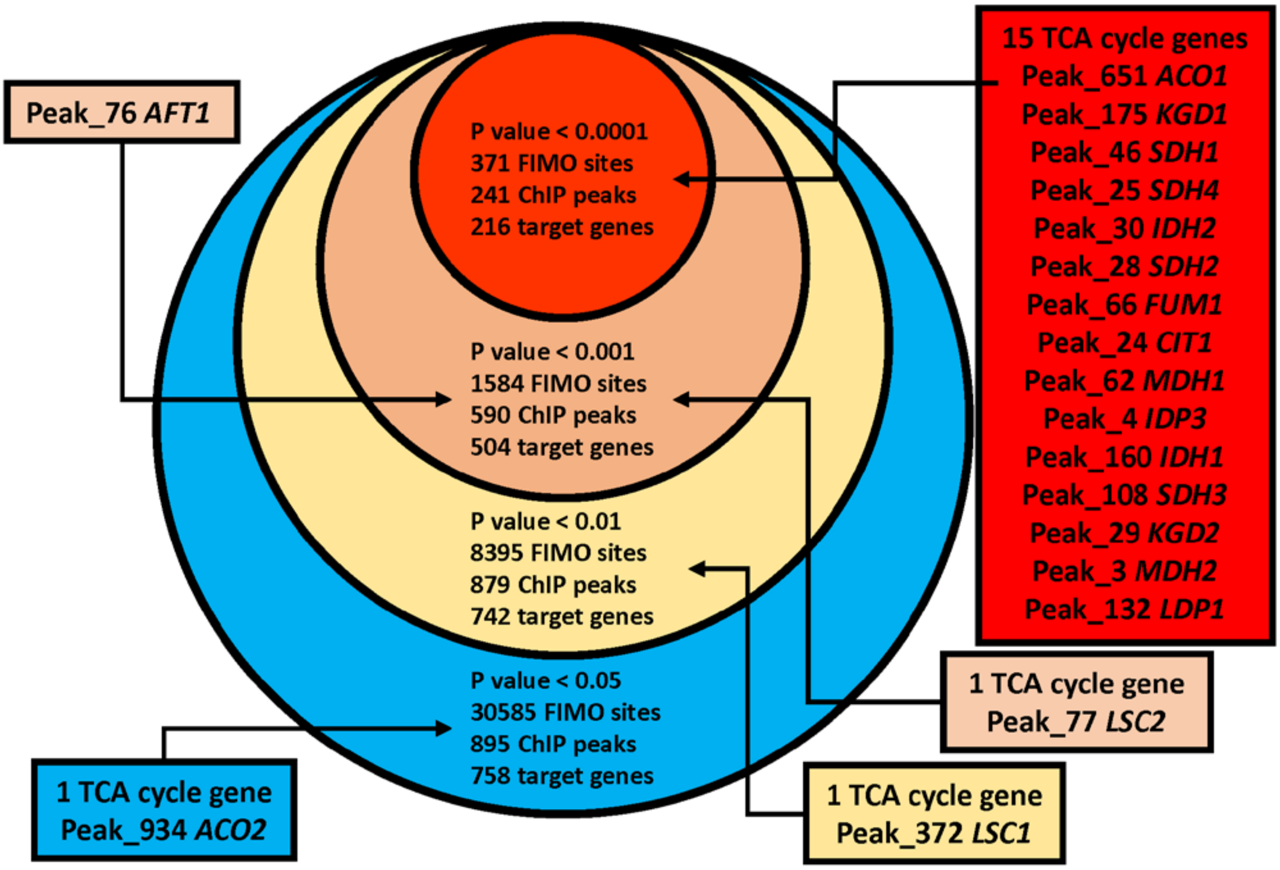

Figure S4. Motif re-scanning for *L. kluyveri* Sef1 target genes. Scanning of 895 ChIP peaks for occurrences of the Lk-Sef1 binding motif. Scanning results (numbers of peaks and motif sites) sorted according to different p-value cut-offs (<0.05 to <0.0001) are shown in the stacked Venn diagram. The distributions of 18 peaks targeting TCA cycle genes and 1 peak targeting the iron-responsive regulator *AFT1* are indicated on the diagram.

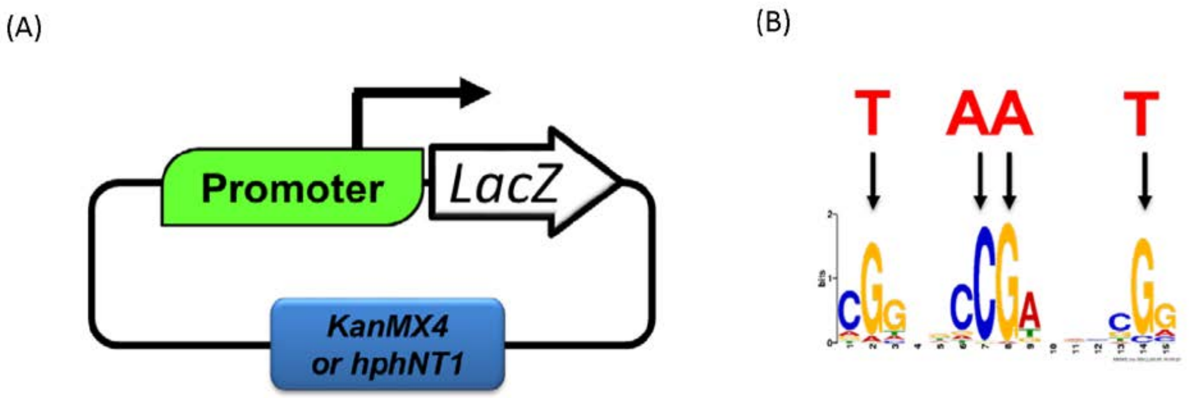

Figure S5. Design of the verification system for Sef1 motif prediction by the LacZ reporter-based promoter assay. (A) The plasmid-based LacZ reporter system. The candidate promoters were cloned upstream of a *LacZ* gene into the yeast centromeric plasmid. The reporter plasmids carry either a G418 resistance marker or an HGB resistance marker. (B) Loss-of-function mutation manipulation of the Lk-Sef1 binding motif. Three consensus Gs and one consensus C were mutated to A or T as shown.

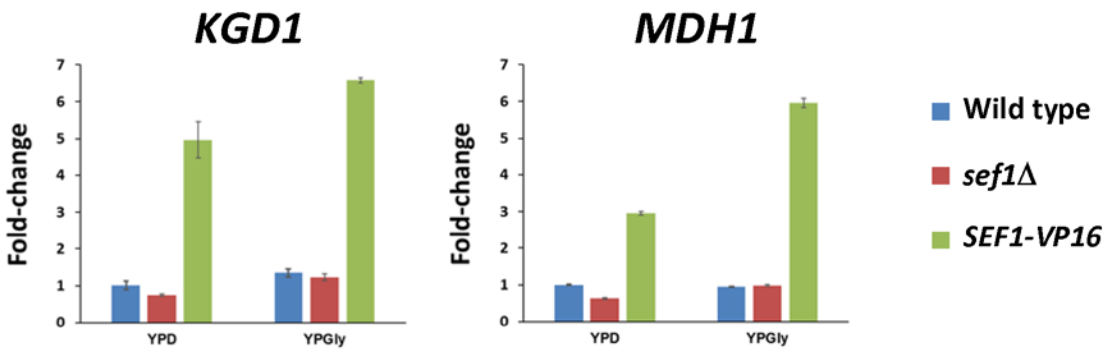

Figure S6. Gene expression verification for *L. kluyveri* Sef1 direct target genes. RNA expression from the *L. kluyveri* wild-type, *sef1*Δ, and *SEF1-VP16* cells grown to early log-phase under both YPD and YPGly conditions were compared by means of qPCR. Two *sef1*Δ-insensitive target genes (*KGD1* and *MDH1*) were assayed. The relative fold-change of each gene is shown as $2^{-\Delta\Delta CT}$, using *ACT1* as the endogenous control and the ΔC_T value from the wild-type sample in YPD as the corresponding calibration value. Expression levels are displayed as means ± standard deviation from at least three technical repeats.

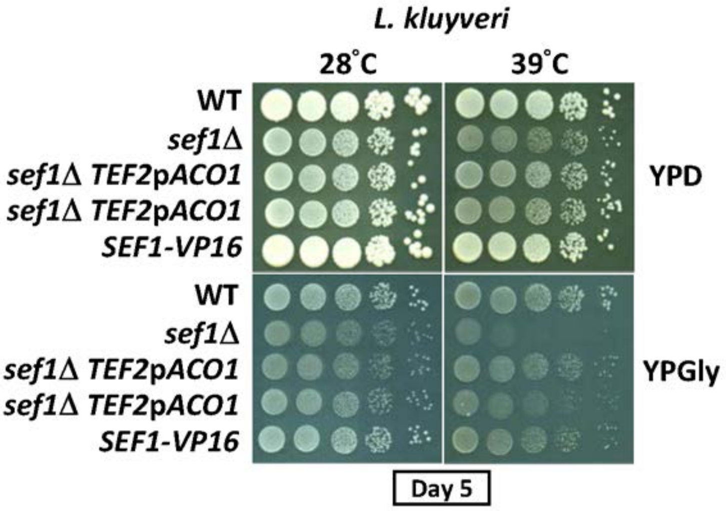

Figure S7. Overexpression of *ACO1* in the *Lk-sef1*Δ mutant. Overexpression of *ACO1* via the *TEF2* promoter (SAKL0B08294g) partially restored the fitness of the *Lk*-*sef1*Δ mutant. The plates were incubated at indicated temperatures for 5 days.

(A)

SEF1-VP16 vs WT

Systemic name	Gene	YPD	dexEnrich	YPGly	glyEnrich
SAKL0C07502g	*GPD1*		58.1	1.58	128
SAKL0G03498g	*UBA2*		40.1	1.56	132
SAKL0H19690g	*ACH1*		21.4	1.56	119
SAKL0E01650g	*MIC17*	1.97	74.5		14.5
SAKL0D11484g	*SPS19*	1.77	20.1	1.6	55.5
SAKL0B05940g	NA	1.72	16.9	3.34	49
SAKL0H07238g	*ISU1*	1.66	12.4	1.82	39.5
SAKL0G06842g	*HSP10*		9.15	2.19	17.7
SAKL0F00682g	*BRR6*	1.56	5.39	1.84	21.2
SAKL0A00616g	YCR087C-A		5.62	3.31	18.2
SAKL0F09108g	*ALD5*	1.63	14.7		8.18
SAKL0H23892g	*MET17*	1.52	10.4	2.01	9.97
SAKL0H11000g	*AQY1*	1.51	10.9		9.35
SAKL0F03718g	*YLH47*	1.73	13.4	1.6	6.04
SAKL0H24442g	*AGC1*	1.55	5.12	2.26	13.8
SAKL0F15048g	*ECM13*		3.91	1.62	12.4
SAKL0H17226g	*TRS85*		2.63	2.11	13.2
SAKL0D13222g	*CLU1*		5.87	1.64	9.92
SAKL0B03212g	*ICL2*		N.D.	1.94	14.6
SAKL0A07392g	*AME1*		2.89	3.18	10.7
SAKL0F14784g	*FRE3*	1.84	N.D.		9.82
SAKL0D15334g	NA	1.91	N.D.	4.02	9.31
SAKL0H23210g	*EAT1*		N.D.	2.53	8.69
SAKL0D06908g	*SDH8*	1.82	3.26	2.12	5.95
SAKL0D06886g	*BNA3*		3.26	1.58	5.95
SAKL0G14762g	*UGA4*	2.84	N.D.	6.49	7.19
SAKL0H25960g	*RPL8A*	1.53	3.6		4.54
SAKL0B06028g	YMR244W		2.1	1.53	3.53
SAKL0C01276g	YDR514C		1.57	1.64	3.89
SAKL0C08426g	*SDH5*		N.D.	2.23	4.45
SAKL0F14718g	*FRE3*	2.14	N.D.		4.41
SAKL0A04532g	*AFT1*	1.81	N.D.	1.63	4.28
SAKL0E12584g	*FMP41*		N.D.	1.52	4.12
SAKL0F02904g	YGR126W		N.D.	1.7	3.85
SAKL0E01760g	*RCL1*	1.62	2.52		2.23
SAKL0A10538g	NA		N.D.	1.69	3.55
SAKL0D11506g	*BUD16*	1.57	N.D.		3.26
SAKL0F02376g	*BTN2*	1.57	2.3		1.77
SAKL0D08646g	*PET8*		N.D.	1.51	2.85
SAKL0D02046g	YMR265C	1.53	N.D.	2.03	2.46
SAKL0B03322g	*FTR1*	1.86	N.D.	1.6	2.2
SAKL0A09306g	*LEU5*		N.D.	2.5	1.73
SAKL0C10428g	*BRX1*		N.D.	1.75	1.41
SAKL0D03036g	*RSM10*		1.27	1.57	N.D.
SAKL0H24178g	*CRG1*		N.D.	1.6	1.18

Up ≥2X

Up ≥1.5X

Down ≥1.5X

<1.5X

(B)

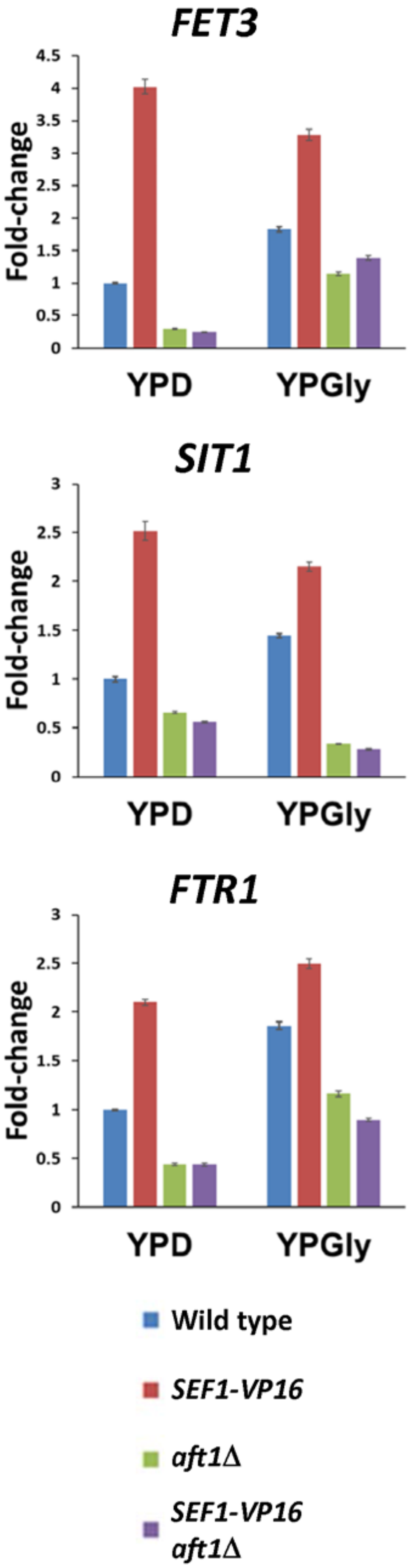

Figure S8. Differential expression of non-TCA cycle genes in response to the gain-of-

function *SEF1-VP16* mutation. (A) RNA expression profiles from the *L. kluyveri* wild-type and *SEF1-VP16* cells grown to early log-phase under both YPD and YPGly conditions were compared. Upregulated genes with ≥ 1.5 fold-change are highlighted in red. Downregulated genes in *SEF1-VP16* with ≥ 1.5 fold-change are highlighted in green. Non-differentially-expressed genes (<1.5 fold-change) are marked in black. Non-TCA cycle genes directly targeted by Sef1 and differentially expressed under at least either one of the conditions are shown in the table. For comparison, the ChIP enrichment values (from wild-type Sef1 ChIP-seq data, Table S3) of peaks that target each gene under the YPD and YPGly conditions (dexEnrich and glyEnrich, respectively) are also included in the table. The genes are ranked in descending order from top to bottom according to the sum of dexEnrich and glyEnrich (highlighted by the intensity of blue color). Peaks not called are labeled “N.D.”. The *AFT1* gene is highlighted in pink. (B) RNA expression from the *L. kluyveri* wild-type, *SEF1-VP16*, *aft1*Δ, and *SEF1-VP16* / *aft1*Δ cells grown to early-mid log-phase under both YPD and YPGly conditions in the absence (iron-rich) or presence (iron-deprived) of an iron chelator (200 μM BPS). Gene expression of three *AFT1* target genes related to iron-uptake (*FET3*, *SIT1*, *FTR1*) was measured by using qPCR. Upregulation of all three genes by Sef1-Vp16 depends on Aft1. The relative fold-change for each gene is shown as $2^{-\Delta\Delta CT}$, by using *ACT1* as endogenous control and the ΔC_T value from the wild-type sample in YPD as the corresponding calibration value. Expression levels are displayed as means ± standard deviation from at least three technical repeats.

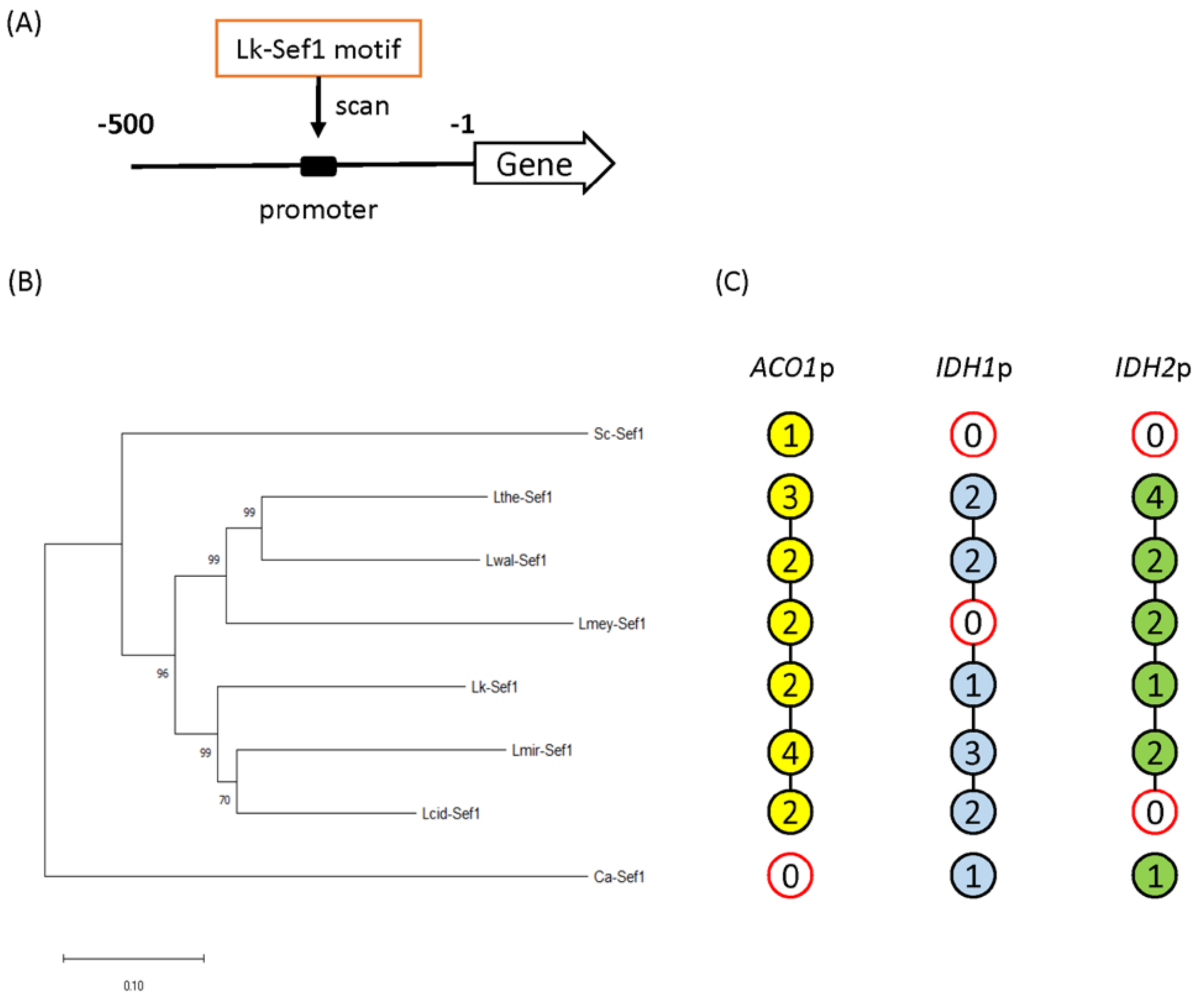

Figure S9. Lk-Sef1 motif scanning for TCA cycle genes in multiple *Lachancea* species. (A) Scanning of 500-bp (-1 to -500) promoters for occurrences of the Lk-Sef1 binding motif by FIMO. (B) Scanning results (numbers of high confident motif sites identified according to the p-value cut-off (<0.0001) are shown. Three promoters of representative TCA cycle genes (*ACO1*, *IDH1*, and *IDH2*) were chosen for analysis. The *Lachancea* Sef1 orthologous sequences were aligned in MUSCLE, and a phylogenetic tree was built using the NJ algorithm with bootstrap support values from 1000 replicates. The optimal tree with the sum of branch length = 1.98609084 is shown. The percentage of replicate trees in which the associated taxa clustered together in the bootstrap test is shown next to the branches. This

analysis involved 8 amino acid sequences. There were a total of 1226 positions in the final dataset. Alignments and evolutionary analyses were conducted in MEGA X. The *C. albicans* and *S. cerevisiae* were used as the outgroups (Lthe: *Lachancea thermotolerans*; Lwal: *Lachancea waltii*; Lmey: *Lachancea meyersii*; Lmir: *Lachancea mirantina*; Lcid: *Lachancea cidri*) (Vakirlis, et al. 2016).

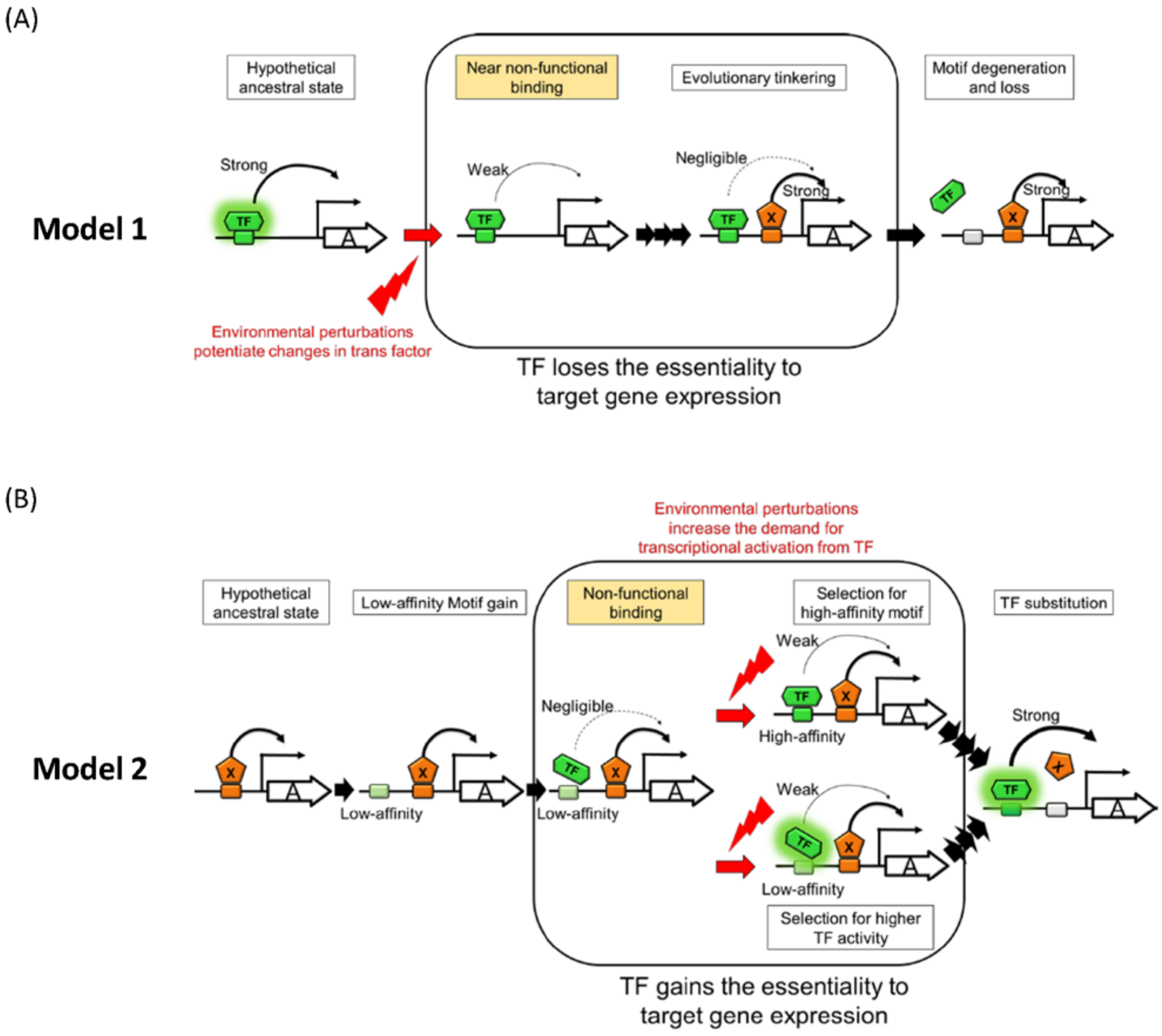

Figure S10. Proposed models for the contribution of non-functional transcription binding to transcriptional rewiring. (A) Model 1：Evolutionary tinkering model for the loss of an old transcription factor. (B) Model 2：Nonfunctional-to-functional binding model for the gain of a new transcription factor.

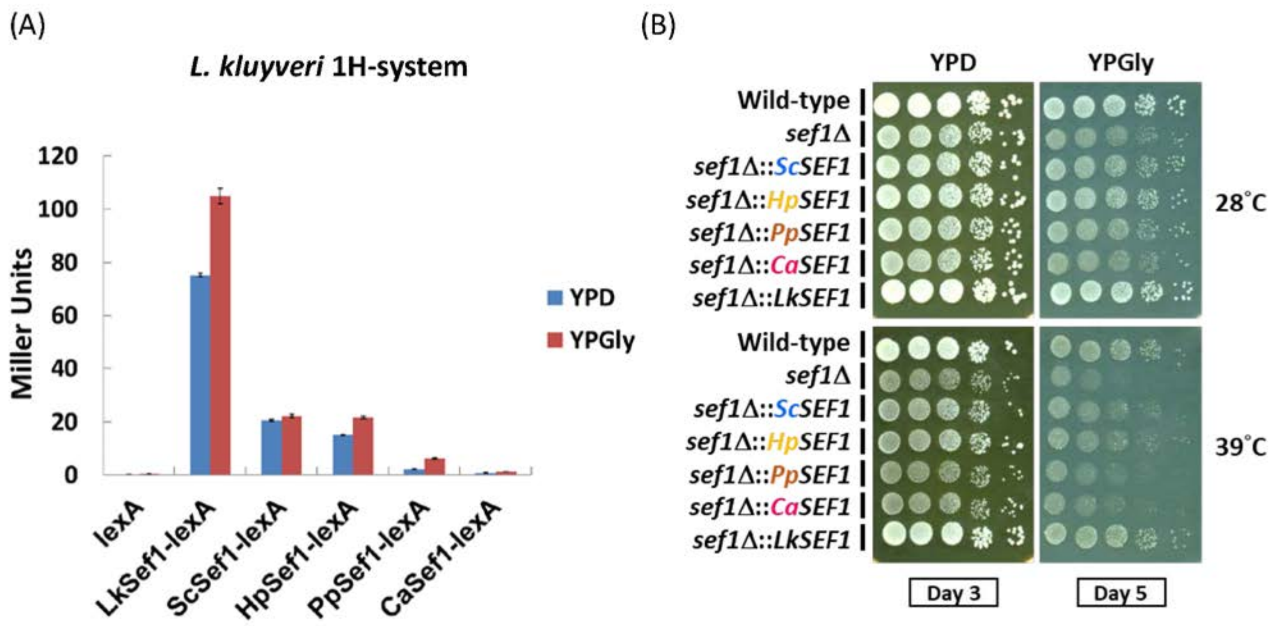

Figure S11. Transcriptional activation activities of Sef1 orthologs correlate with the complementation activities. (A) One-hybrid assays for Sef1 orthologs in *L. kluyveri* using the C-terminally fused LexA system. LacZ activity was measured using the liquid-galactosidase assay and displayed as average Miller units ± S.D. from at least three technical repeats. (B) Complementation of *Lk-sef1*Δ with other *SEF1* orthologs using spot assays: each *SEF1* ortholog was put into the *Lk-SEF1* locus under the control of native the *Lk-SEF1* promoter and terminator; *L. kluyveri* (Lk), *S. cerevisiae* (Sc), *H. polymorpha* (Hp), *P. pastoris* (Pp), *C. albicans* (Ca).

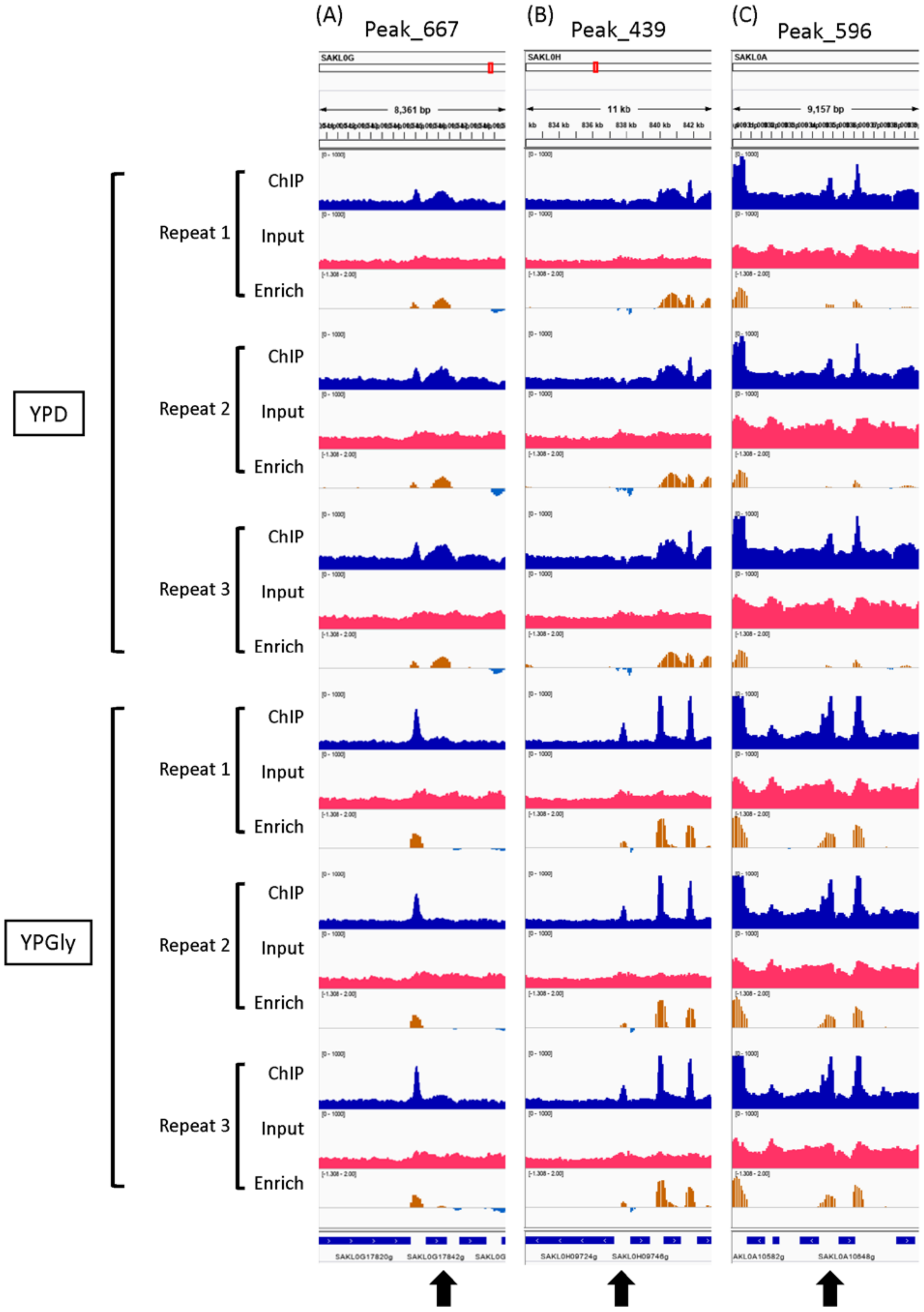

Figure S12. Examples of the reproducible weak (low-enrichment) peak identification.

(A) Peak_667：YPD-only binding peak. (B) Peak_439：YPGly-only binding peak. (C) Peak_596: both binding peak. The arrows indicate the position of each peak. The image was generated using IGV (Robinson, et al. 2011).

Table S1. Transcriptional profiles of iron-responsive genes in the wild type of *L. kluyveri*.

Table S2. Transcriptional profiles of differentially expressed genes in the *sef1*Δ or *aft1*Δ mutants under the low-iron condition compared to wild-type *L. kluyveri*.

Table S3. Complete ChIP-seq results of *L. kluyveri* Sef1.

Table S4. Differentially expressed gene profiles in the log-phase *sef1*Δ mutant in YPD.

Table S5. Differentially expressed gene profiles in the log-phase *sef1*Δ mutant in YPGly.

Table S6. Differentially expressed gene profiles in the log-phase gain-of-function *SEF1-VP16* mutant in YPD.

Table S7. Differentially expressed gene profiles in the log-phase *SEF1-VP16* gain-of-function mutant in YPGly.

Table S8. Strains list.

Table S9. Plasmid list.

Table S10. Primer list.

Table S11. Genome resources.

Table S12. Media and important chemicals.

Table S13. Best probe list for *L. kluyveri* microarray analyses.

Table S14. Cross-reference table for *L. kluyveri*-to-*S. cerevisiae* ortholog conversion.

Data File S1. Raw results of MEME-ChIP analysis for *L. kluyveri* Sef1 peaks.

(Due to the limitation of the journal, the data file S1 is available only upon request.)

Data File S2. Raw files of FIMO results for *L. kluyveri* Sef1 peaks.

(Due to the limitation of the journal, the data file S2 is available only upon request.)

Data File S3. Raw files of FIMO results for *Lachancea ACO1*, *IDH1*, and *IDH2* promoters

(Due to the limitation of the journal, the data file S3 is available only upon request.)
